# Spatiotemporal Gaussian representation-based dynamic reconstruction and motion estimation framework for time-resolved volumetric MR imaging (DREME-GSMR)

**Published:** 2026-04-07

**Authors:** Jiacheng Xie, Hua-Chieh Shao, Can Wu, Ricardo Otazo, Jie Deng, Mu-Han Lin, Tsuicheng Chiu, Jacob Buatti, Viktor Iakovenko, You Zhang

**Affiliations:** 1 The Advanced Imaging and Informatics for Radiation Therapy (AIRT) Laboratory, Dallas, TX 75390, United States of America; 2 The Medical Artificial Intelligence and Automation (MAIA) Laboratory, Dallas, TX 75390, United States of America; 3 Department of Radiation Oncology, University of Texas Southwestern Medical Center, Dallas, TX 75390, United States of America; 4 Department of Medical Physics, Memorial Sloan Kettering Cancer Center, New York, NY 10065, United States of America

**Keywords:** Motion management, Image reconstruction, Time-resolved MRI, Real-time imaging, Gaussian representation, Motion modeling

## Abstract

Time-resolved volumetric MR imaging that reconstructs a 3D MRI within sub-seconds to resolve deformable motion is essential for motion-adaptive radiotherapy. Representing patient anatomy and associated motion fields as 3D Gaussians, we developed a spatiotemporal Gaussian representation-based framework (DREME-GSMR), which enables time-resolved dynamic MRI reconstruction from a pre-treatment 3D MR scan without any prior anatomical/motion model. DREME-GSMR represents a reference MRI volume and a corresponding low-rank motion model (as motion-basis components) using 3D Gaussians, and incorporates a dual-path MLP/CNN motion encoder to estimate temporal motion coefficients of the motion model from raw k-space-derived signals. Furthermore, using the solved motion model, DREME-GSMR can infer motion coefficients directly from new online k-space data, allowing subsequent intra-treatment volumetric MR imaging and motion tracking (real-time imaging). A motion-augmentation strategy is further introduced to improve robustness to unseen motion patterns during real-time imaging. DREME-GSMR was evaluated on the XCAT digital phantom, a physical motion phantom, and MR-LINAC datasets acquired from 6 healthy volunteers and 20 patients (with independent sequential scans for cross-evaluation). DREME-GSMR reconstructs MRIs of a ~400ms temporal resolution, with an inference time of ~10ms/volume. In XCAT experiments, DREME-GSMR achieved mean(s.d.) SSIM, tumor center-of-mass-error(COME), and DSC of 0.92(0.01)/0.91(0.02), 0.50(0.15)/0.65(0.19) mm, and 0.92(0.02)/0.92(0.03) for dynamic reconstruction/real-time imaging. For the physical phantom, the mean target COME was 1.19(0.94)/1.40(1.15) mm for dynamic/real-time imaging, while for volunteers and patients, the mean liver COME for real-time imaging was 1.31(0.82) and 0.96(0.64) mm, respectively. Compared with a state-of-the-art method (DREME-MR), DREME-GSMR reduced training time by 30%, enhancing the applicability of dynamic and real-time MRI imaging for potential clinical translation. Code.

## 1. Introduction

In radiotherapy, magnetic resonance imaging (MRI) provides superior soft tissue contrast for anatomical visualization without exposing patients to ionizing radiation. For more accurate tumor targeting and dose delivery, MRI has been widely integrated into clinical workflows to enable MRI-only treatment planning (Greer et al., 2019; Owrangi et al., 2018) and MRI-guided radiotherapy (Corradini et al., 2019; Hall et al., 2019; Owrangi et al., 2018). For anatomical sites with respiration-induced motion (Bertholet et al., 2016; Seppenwoolde et al., 2002), especially the thoracic and abdominal regions, four-dimensional MRI (4D-MRI) was developed for motion management (Stemkens et al., 2018). During a typical 4D-MRI acquisition, an external or internal respiratory surrogate signal is used to sort continuously acquired k-space data into predefined respiratory-phase bins and reconstruct volumetric MR images from each bin, yielding a motion-resolved sequence of three-dimensional images that spans a representative breathing cycle (Menchón-Lara et al., 2019; Rajiah et al., 2023; Stemkens et al., 2018). By analyzing patient-specific breathing patterns on 4D-MRIs, clinicians can select tailored motion-mitigation strategies, such as breath-hold techniques or respiratory gating, to reduce target localization uncertainty (Ball et al., 2022; Paganelli et al., 2018). Despite its utility, 4D-MRI has inherent limitations. To obtain sufficient data for every respiratory phase, the scanner must sample the patient’s anatomy over multiple breathing cycles, increasing the likelihood of intra-scan motion variability that manifests as image artifacts and inconsistencies. The phase-sorting process assumes strictly periodic and reproducible breathing, an assumption that often fails in the presence of irregular motion patterns (e.g., amplitude variations, frequency shifts, baseline drifts), leading to mis-sorted data and degraded image quality.

To overcome these shortcomings, *time-resolved* *dynamic* volumetric MRI retrospectively reconstructs a sequence of three-dimensional images at a high temporal resolution (Nayak, 2019), without using external or internal surrogates for gating or sorting. Each frame is reconstructed from a highly undersampled subset of k-space data (e.g., tens of radial spokes), effectively freezing the anatomy during a short acquisition window and eliminating intra-frame motion. Recent single-scan-based frameworks such as MR-MOTUS (Huttinga et al., 2021a; Huttinga et al., 2020), Extreme MRI (Ong et al., 2020), and STINR-MR (Shao et al., 2024) jointly reconstruct all time-frames by exploiting global spatiotemporal correlations, thereby addressing the extreme undersampling issue. However, because these methods require a complete dataset for joint optimization, they cannot provide volumetric images in ‘true’ real time during radiation delivery. To enable real-time treatment verification and adaptation, *time-resolved* *real-time* volumetric MR imaging (Bertholet et al., 2019; Lombardo et al., 2024; Nayak et al., 2022) is required to capture instantaneous anatomical motion variations during radiation delivery in sub-seconds (<500 milliseconds for respiratory motion) (Keall et al., 2021), including both image acquisition and reconstruction time, demanding extremely efficient reconstruction and tracking algorithms from highly undersampled data.

With advances in deep learning (DL) and high-performance GPU computing, numerous DL-based methods have been proposed for fast MRI reconstruction and motion tracking. Some techniques directly generate high-quality MR images from undersampled k-space (Yang et al., 2017; Zhu et al., 2018), whereas others perform de-aliasing or denoising in the image domain (Huang et al., 2022; Liu et al., 2022; Schlemper et al., 2017). Although fast, these reconstruction-based approaches are predominantly two-dimensional and require additional segmentation for target localization, which can introduce further uncertainties and latency. Registration-based DL approaches (Hunt et al., 2023; Shao et al., 2022; Terpstra et al., 2020; Wei et al., 2023) enable three-dimensional motion estimation under severe undersampling. However, most registration-based methods incorporate patient-specific prior images or motion models, which can bias motion estimation when the anatomy/contrast and the motion model captured from the prior images are outdated. Large-scale, high-quality MR datasets are also required for training the DL models, limiting generalizability to out-of-distribution scenarios. To address these challenges, the Dynamic Reconstruction and Motion Estimation for MRI (DREME-MR) framework was proposed (Shao et al., 2025b). Based on the STINR-MR (Shao et al., 2024) framework on dynamic MRI reconstruction and the DREME (Shao et al., 2025a) framework on X-ray-based real-time imaging, DREME-MR unifies dynamic MRI reconstruction and real-time motion estimation within a single, ‘one-shot’ training framework. Validated on digital phantoms and human data, DREME-MR achieved 3 mm spatial and 100-150 ms temporal resolutions for cardiorespiratory motion without patient-specific priors or phase binning. However, its major drawback is training time (~ 200 min on an NVIDIA Tesla V100), which limits clinical deployment. In addition, its real-time imaging capability in a human subject was evaluated by using 75% of the acquired data for training and the remaining 25% for testing. As a result, the reported performance primarily reflects the model’s ability to generalize within the same acquisition (intra-fraction) rather than its generalizability in a truly real-time setting, where training and inference data should be collected sequentially and independently. Consequently, its evaluation remains relatively narrow, and the robustness of the learned model under substantial discrepancies between pretreatment motion patterns and in-treatment motion has not been fully evaluated. This is a critical limitation for real-time imaging, where motion characteristics may vary considerably over the course of treatment. Furthermore, the MRI data used in DREME-MR were acquired with a 3D golden-mean Koosh-ball radial trajectory, which has mainly been reported in research or prototype implementations rather than as a routine clinical MR-LINAC acquisition (Feng, 2022; Huttinga et al., 2021b). In contrast, current clinical MR-LINAC workflows more commonly rely on stack-of-stars radial sequences for motion-impacted sites like the abdomen (Feng, 2022; Keesman et al., 2024; Ferris et al., 2024; Daly et al., 2024; Paulson et al., 2020), which may limit the immediate translational relevance of the framework.

In this study, we propose DREME-GSMR, which introduces the strong representation power of Gaussians into the dynamic and real-time MRI pipeline to address the computation-intensive and real-time imaging robustness issues of DREME-MR. DREME-GSMR consists of three key components: (i) a dense 3D Gaussian set to reconstruct a reference-frame MRI, (ii) another Gaussian set to model intra-scan motion through three-level, coarse-to-fine motion-basis components (MBCs) to capture voxelwise motion pattern variations, (iii) and a dual-path multilayer perceptron (MLP)/CNN-based motion encoder that computes temporal coefficients for these MBCs from multi-coil k-space signals. The coefficient-scaled MBCs are combined into deformation vector fields (DVFs) to deform the reference MRI into time-resolved MRIs. DREME-GSMR enables dual-task learning in a single training session: (1) dynamic volumetric MRI reconstruction, which reconstructs a sequence of volumetric MR images from a pretreatment 3D MR scan by jointly optimizing a Gaussian-based reference image and a motion model; and (2) real-time imaging and motion tracking, which trains a motion encoder for the motion model to allow real-time image/motion inference on newly acquired k-space data. The main contributions of this work are summarized as follows:

- We propose a 3D Gaussian representation-based framework, DREME-GSMR, for time-resolved dynamic and real-time volumetric MR imaging. By jointly solving a reference image and a motion model with 3D Gaussians, the framework achieves high-quality reconstruction and accurate motion estimation while reducing training time by 30% compared with prior approaches.
- We developed an MLP-CNN dual-path motion encoder with motion-augmentation training to enable robust real-time motion estimation beyond the training distribution, overcoming limitations of the MLP-only encoder in DREME-MR.
- We extensively evaluated the proposed framework on digital phantom simulations, physical phantom measurements, and clinical datasets, including 6 healthy volunteers and 20 patients. All measured data were acquired using a clinically available golden-angle stack-of-stars (SOS) trajectory on an MR-LINAC system. Importantly, each clinical case contains two sequential scans, allowing training and testing to be performed on independent acquisitions for cross-evaluation and providing a more realistic assessment of real-time imaging performance in an online adaptive radiotherapy setting.

## 2. Related work

### 2.1 Time-resolved Dynamic volumetric MRI reconstruction

Dynamic MRI reconstruction seeks to reconstruct a time-resolved sequence of MR volumes from highly limited k-space data per volume, thereby avoiding motion sorting while achieving the temporal resolution needed to capture anatomical changes (Nayak, 2019). This makes it a highly ill-posed spatiotemporal inverse problem, since volumetric images must be recovered from severely undersampled k-space data. Existing approaches have addressed this challenge by exploiting spatiotemporal correlations across dynamic volumes, including model-based motion-estimation methods, factorization-based dynamic representations, and joint reconstruction-registration frameworks.

MR-MOTUS (Huttinga et al., 2021a; Huttinga et al., 2020) proposed a model-based framework for estimating non-rigid 3D motion directly from highly undersampled k-space data, and obtaining dynamic volumes by warping a pre-acquired reference MR image with the estimated deformation fields. It demonstrated that a 250-frame 3D cine MRI could be reconstructed using 30 spokes per frame. However, its performance is inherently dependent on the quality and consistency of the reference image acquired in a separate scan, where intensity discrepancies unrelated to anatomical deformation between the reference and dynamic acquisitions would impact motion estimation accuracy (Zhang et al., 2017). Extreme MRI (Ong et al., 2020) uses multiscale low-rank matrix factorization to model volumetric dynamics from continuous non-gated MRI acquisitions. It enables high-resolution dynamic MRI reconstruction while preserving transient and non-periodic motion that is often lost in gating-based methods. Nevertheless, the method remains computationally demanding and is prone to temporal flickering artifacts (Ong et al., 2020; Shao et al., 2025b). More importantly, subsequent evaluations suggest that, while it is effective in capturing coarse-scale contrast variations associated with large organ motion, it is less capable of resolving fine-scale anatomical variations, such as small lung tumors, thereby leading to substantial localization error (Shao et al., 2025b). A more recent work, STINR-MR, addresses the highly ill-posed 3D dynamic MRI reconstruction problem through a joint image reconstruction and deformable motion estimation framework. In this approach, the reference-frame 3D MR image is represented by a spatial implicit neural representation (INR), while the dynamic motion is modeled by a temporal INR that maps input time points to weighting coefficients for a principal component analysis (PCA)-based motion model derived from a prior or onboard 4D-MRI set. The resulting weighted motion bases are used to generate time-resolved DVFs, which deform the reference image to recover the dynamic image sequence. By jointly refining both the reference image and the motion representation from undersampled MRI data, STINR-MR enables 3D dynamic MR reconstruction with a high temporal resolution (<100 ms) and a 3 mm isotropic spatial resolution. This framework overcomes key limitations of earlier methods, such as MR-MOTUS and Extreme MRI, and demonstrates state-of-the-art (SOTA) performance in volumetric dynamic MRI reconstruction and motion estimation. However, it still relies on a low-dimensional PCA motion model prior, which may limit its flexibility/accuracy under highly irregular anatomical motion.

Overall, although these methods have substantially advanced dynamic volumetric MRI reconstruction, existing approaches remain limited by dependence on reference images or predefined motion priors, insufficient sensitivity to fine-scale anatomical variation, and high computational cost, all of which present challenges for robust clinical applications.

### 2.2 Real-time volumetric MR imaging

Although dynamic volumetric MRI provides richer motion information than conventional, sorting-based 4D-MRI, existing reconstruction methods are not designed for beam-on applications because they typically require access to the full acquisition sequence and rely on computationally intensive spatiotemporal modeling or prior-driven optimization. To enable real-time treatment verification and adaptation (Keall et al., 2025; McNair and Buijs, 2019; Keall et al., 2019), time-resolved real-time MR imaging is needed to capture anatomical changes with a sub-second latency (Bertholet et al., 2019; Nayak et al., 2022; Lombardo et al., 2024). For respiratory motion management, the recommended end-to-end latency, including both image acquisition and reconstruction, is within 500 milliseconds (ms) (Keall et al., 2021). This stringent requirement largely precludes the use of time-consuming dynamic volumetric MRI reconstruction strategies. Moreover, because only limited anatomical information can be sampled within such a short time window due to the inherently low sampling efficiency of MRI, highly efficient reconstruction and motion-tracking algorithms are required.

Existing methods for real-time volumetric MR imaging can be broadly categorized into DL-based registration approaches, offline-online motion-model methods, and dual-task learning methods. Among DL-based approaches, TEMPEST (Terpstra et al., 2021) estimates 3D motion fields between a high-quality static MR volume and an undersampled dynamic volume using a multi-resolution pyramid registration framework, achieving registration accuracy below 2 mm with a latency under 200 ms. However, as a supervised method, it requires “ground-truth” 3D motion fields for training, which are typically generated by other registration techniques such as optical flow. As a result, errors in the training labels may propagate to the network and introduce intrinsic bias. To mitigate this limitation, unsupervised models have been developed for real-time motion estimation. Based on the VoxelMorph (Balakrishnan et al., 2019) architecture, KS-RegNet (Shao et al., 2022) was proposed to learn deformable registration through k-space data consistency, by comparing the reprojected k-space of the registered images with the acquired undersampled k-space data, thereby avoiding the need for paired motion-field supervision. This method achieved target localization accuracy below 2 mm, although the latency remained around 600 ms due to the use of non-uniform Fourier transforms. Similarly, Wei et al. registered a prior 3D MR image to onboard coronal 2D MR images to generate updated volumetric MR images in real time, reporting a localization error below 2.6 mm with a latency under 100 ms (Wei et al., 2023). However, these approaches typically incorporate patient-specific prior information to improve localization accuracy, which may introduce bias into motion estimation because the anatomy and motion patterns can change over the course of treatment. In addition, the performance of these DL-based methods may degrade when applied to out-of-distribution data, as large-scale MR datasets capturing diverse motion patterns remain limited for training.

Another line of work adopts an offline-online strategy. The original MR-MOTUS framework (Huttinga et al., 2021a; Huttinga et al., 2020) was later extended by the same group to real-time imaging (Huttinga et al., 2022). In the offline phase, the extended MR-MOTUS iteratively reconstructs a 10-phase 4D-MRI from a 10-minute MR acquisition and derives a B-spline-based motion model. In the online phase, it uses the learned anatomy and motion model to estimate real-time motion fields from a 67 ms MR acquisition, achieving a total latency of 170 ms. More recently, MRSIGMA (Feng et al., 2020), built upon the 4D-MRI reconstruction framework XD-GRASP (Feng et al., 2016), was introduced for real-time volumetric MR imaging. During the offline phase, it constructs a 10-phase 4D motion dictionary that associates MR motion signatures with corresponding motion states. During real-time imaging, it performs signature matching to identify the most likely motion state and recover the corresponding 3D volume. However, because both methods rely on motion-sorted 4D-MRI reconstruction in the offline phase, they inherit the same fundamental limitation as conventional 4D-MRI, namely, a restricted set of motion states that may be insufficient to capture irregular or non-periodic motion.

To address these limitations, DREME-MR (Shao et al., 2025b) was proposed as a dual-task learning framework that integrates dynamic volumetric MRI reconstruction into a real-time imaging setting. Following a ‘one-shot’ learning strategy, DREME-MR does not require large population-based pretraining datasets or patient-specific prior information, as it is trained only on k-space data acquired from a patient-specific pre-treatment MR scan to reconstruct a dynamic MRI sequence without motion sorting/binning, while obtaining a motion model for real-time imaging. The method achieved high spatiotemporal resolution, with a 3 mm spatial resolution and a 100-150 ms temporal resolution. However, its clinical practicality remains limited by the prolonged patient-specific training time, which was reported to be approximately 200 minutes. More importantly, its robustness to discrepancies between the pre-treatment motion used for training and real-time motion has not been comprehensively evaluated, which may restrict performance under variable or irregular motion. In addition, the method has only been validated on data acquired with a 3D golden-angle Koosh-ball radial trajectory, whereas its performance on more clinically common sampling strategies, such as SOS, has not yet been demonstrated.

Overall, although substantial progress has been made toward real-time volumetric MR imaging, existing methods remain constrained by dependence on prior anatomy or motion models, limited ability to capture irregular motion, potential generalization issues, and patient-specific computational overhead. These limitations continue to hinder robust and practical deployment for real-time treatment verification and adaptation during beam-on.

### 2.3 3D Gaussian representation

Recently, a machine learning technique named 3D Gaussian representation (Fei et al., 2024) has been applied in medical image reconstruction for X-ray-based CT/CBCT (Zha et al., 2024) and MRI reconstruction (Peng et al., 2025a; Wu et al., 2024). In this approach, anatomy is represented as a collection of 3D Gaussian functions instead of dense voxel grids or neural networks, preserving the continuous volumetric properties of anatomy while avoiding unnecessary computations in empty spaces. It has demonstrated significant advantages over implicit neural representation (INR)-based methods in both reconstruction speed and accuracy for both CT/CBCT (Li et al., 2025; Lin et al., 2024; Zha et al., 2024) and MRI (Wu et al., 2024) reconstruction tasks. Gaussian-based methods employ explicit basis functions, Gaussian primitives, to directly represent the target volume, offering a greater capacity to preserve high-frequency anatomical details such as bony edges. For example, PMF-STGR (Xie et al., 2025) employed 3D Gaussians to represent both the reference image and motion bases in dynamic CBCT reconstruction, resulting in an approximately 50% reduction in reconstruction time and memory consumption compared with INR-based formulations. These advantages have also motivated the extension of Gaussian-based representations from static image reconstruction to motion-resolved MRI. In this direction, MoRe-3DGSMR (Peng et al., 2025b) reconstructs 4D-MRIs by firstly reconstructing a reference image of a motion state using a Gaussian representation and then warping it with DVFs resolved from a convolutional neural network to generate volumes of the remaining motion states. While achieving good reconstruction accuracy, it depends on phase sorting and binning with limited temporal resolution, which fails to capture irregular breathing patterns and hinders time-resolved MRI reconstruction. Consequently, it remains far from meeting the requirements for dynamic and real-time MRI imaging.

## 3. Methods

### 3.1 Dynamic and real-time MR imaging overview

In this work (DREME-GSMR), the aim of dynamic MRI reconstruction is to reconstruct a sequence of dynamic images to visualize the anatomical motion from a pre-treatment scan for treatment guidance. As a highly ill-posed spatiotemporal inverse problem, reconstructing dynamic MRIs typically involves solving more than $𝒪(10^{9})$ unknowns (Huttinga et al., 2021a). To simplify the inverse problem, we decoupled it into a joint reconstruction and registration approach with a low-rank motion model, by solving a reference-frame MRI $I_{\mathrm{ref}}(x)$ and the intra-scan, per-frame motion with respect to $I_{\mathrm{ref}}(x)$. A frame here is defined as an MR volume with a sufficient temporal resolution such that the anatomical state captured by each frame is ‘frozen’ with negligible movement. The de-coupling strategy assumes that anatomies captured by each frame are highly correlated with the same underlying anatomy during the scan, which is generally true considering the short scanning time (i.e., ≲ 8 minutes). Accordingly, we leveraged the temporal correlation between frames by assuming that the observed variations in voxel intensity across frames are primarily due to anatomical motion. Therefore, by deforming the reference-frame MRI $I_{\mathrm{ref}}(x)$ with a sequence of time-dependent DVFs d(x,t) that represents the anatomical motion at frame t, the dynamic MRI sequence I(x,t) can be obtained as:

(1) $$I(x,t)=I_{\text{ref}}(x+d(x,t)).$$

where $x\in R^{3}$ denotes the voxel coordinates of the reconstruction volume and t denotes the frame index of the dynamic sequence. This registration-based approach assumes that MR signals acquired at different time points satisfy steady-state conditions and preserve local spin magnetization (Huttinga et al., 2020). In other words, it assumes that the tissue contrast and underlying signal characteristics do not vary significantly between frames, enabling reliable spatial alignment. As a result, it is not suitable for scenarios involving temporal image contrast changes, such as dynamic contrast-enhanced MRI (Padhani, 2002), or for acquisitions performed under non-stationary conditions (Tippareddy et al., 2021). For further dimension reduction to address the ill-posed problem, the time-dependent motion field d(x,t) can be approximated by a low-rank function (Li et al., 2011; Stemkens et al., 2016; Zhang et al., 2007) via a summation of products of spatial $e_{i}(x)$ and temporal $w_{i}(t)$ components:

(2) $$d(x,t)=\sum_{i=1}^{3}w_{i}(t)\times e_{i}(x).$$

The spatial component $e_{i}(x)$ serves as a basis set that spans the motion space, and all motion states of the dynamic sequence can be described by scaling the spatial components via the temporal components $w_{i}(t)$, reducing the number of unknowns to $∼𝒪(10^{7})$. Accordingly, $e_{i}(x)$ and $w_{i}(t)$ are called motion basis components (MBCs) and MBC scores, respectively. Thus, dynamic MRI imaging is equivalent to reconstructing a reference-frame MRI $I_{\mathrm{ref}}$, while determining time-varying MBC scores $w_{i}(t)$ of MBCs $e_{i}(x)$ that capture the underlying anatomical motion. Following the previous study (Li et al., 2011; Shao et al., 2024; Shao et al., 2025b), we used three MBCs (i.e. i=1, 2, 3) for each Cartesian direction to model complex breathing motion.

With the above strategies, the dynamic reconstruction is formulated as an optimization problem:

(3) $$\hat{I}(x,t)=\text{arg}\underset{I_{\text{ref}}(x),w_{i}(t),e_{i}(x)}{\text{min}}\;E\;[{\left\|ℱ[I(x,t)]-s(k,t)\right\|}_{1}]+\lambda R[I(x,t)],$$

where s(k,t) denotes the acquired k-space signal at the frame t, ℱ denotes the operator combining the coil sensitivity encoding and non-uniform fast Fourier transform (NUFFT), ${\left\|\cdot \right\|}_{1}$ denotes the L1 norm, E denotes the expectation value, and λ is the weighting factor of the regularization term R. The first term of Eq. 3 enforces the k-space data consistency by matching the reconstructed k-space data with the acquired k-space signals s(k,t). The second term regularizes the optimization process to mitigate the undersampled reconstruction challenge (see Sec. 3.4.4 for details). After the dynamic reconstruction, the learned reference-frame image $I_{\mathrm{ref}}(x)$ and the correspondingly motion model (i.e., $e_{i}(x)$ and $w_{i}(t)$) would allow DREME-GSMR to infer the MBC scores $w_{i}(t)$ from future limited-sampled k-space acquisitions s(k,t) for real-time imaging (Sec. 3.4.1).

### 3.2 Gaussian representation

We represent the target real-valued objects with a set of learnable 3D Gaussian kernels $G^{3}={\left\{G_{i}^{3}\right\}}_{i=1,\ldots ,M}$ such that each kernel $G_{i}^{3}$ defines a local Gaussian-shaped density field:

(4) $$G_{i}^{3}(x\;|\;\rho_{i},p_{i},\Sigma_{i})=\rho_{i}\cdot \text{exp}\left(-\frac{1}{2}{\left(x-p_{i}\right)}^{T}\Sigma_{i}^{-1}(x-p_{i})\right),$$

where $x\in R^{3}$ denotes the voxel coordinates of the image volume. $\rho_{i}$, $p_{i}\in R^{3}$ and $\Sigma_{i}\in R^{3\times 3}$ are learnable parameters representing the central density, the center location, and the shape size and orientation of each Gaussian point, respectively. The overall density σ(x) at $x\in R^{3}$ can be obtained by summing the densities of kernels, which is the voxelization operation for Gaussians:

(5) $$\sigma (x)=\sum_{i=1}^{M}G_{i}^{3}(x\;|\;\rho_{i},p_{i},\Sigma_{i}).$$

For complex-valued target objects, we represent them with a set of learnable complex-valued 3D Gaussian kernels, where each kernel uses two independent density coefficients ρiRe and ρiIm to model the real and imaginary components, respectively:

(6) $${\tilde{G}}_{i}^{3}(x\;|\;{\rho_{i}}^{Re},{\rho_{i}}^{Im},p_{i},\Sigma_{i})=({\rho_{i}}^{Re}+j{\rho_{i}}^{Im})\cdot \text{exp}\left(-\frac{1}{2}{\left(x-p_{i}\right)}^{T}\Sigma_{i}^{-1}(x-p_{i})\right).$$

The overall complex-valued density can be obtained by:

(7) $$\tilde{\sigma }(x)=\sum_{i=1}^{M}{\tilde{G}}_{i}^{3}(x\;|\;{\rho_{i}}^{Re},{\rho_{i}}^{Im},p_{i},\Sigma_{i}).$$

which can be decomposed into real and imaginary parts as σ~(x)=σRe(x)+jσIm(x).

In DREME-GSMR, both the reference-frame MRI and the MBCs are represented using Gaussians with signed (negative-permitting) density coefficients, enabled by a modified CUDA-based voxelizer adapted from Zha et al. (Zha et al., 2024). Unlike the original non-negative implementation, our signed voxelizer preserves both positive and negative Gaussian contributions during accumulation. For the reference-frame MRI $I_{\mathrm{ref}}$, we use complex-valued 3D Gaussians, where each Gaussian shares the same spatial parameters across channels but has two signed density parameters, to model the real and imaginary components, respectively. In contrast, each MBC Gaussian is defined by its own spatial parameters and a single signed real-valued density coefficient. Thus, the MRI Gaussians differ from the MBC Gaussians in output dimensionality, with the former producing a two-channel complex-valued density and the latter producing a single-channel real-valued density, while both support signed values.

For DREME-GSMR, we used complex-valued 3D Gaussians to represent the reference-frame MRI $I_{\mathrm{ref}}$ to better capture the fine details of the anatomy. In addition, we also used 3D Gaussians to represent the MBCs for the motion model learning, to accelerate the training and avoid over-smoothing.

### 3.3 Stack-of-stars golden-angle radial sampling

In this study, dynamic k-space data were continuously acquired using a stack-of-stars (SOS) golden-angle radial trajectory, which combines radial sampling in the $k_{x}-k_{y}$ plane with Cartesian encoding along $k_{z}$ (Feng, 2022). In SOS acquisition, all spokes at different $k_{z}$ positions acquired at one in-plane angle form a radial stack, and successive stacks are rotated using a golden-angle scheme. This hybrid design inherits the motion robustness and flexible retrospective grouping of radial sampling while preserving the efficiency of Cartesian encoding along $k_{z}$.

Beyond its sampling efficiency, SOS is particularly attractive because it provides intrinsic self-navigation for motion estimation. Prior studies have exploited this property in several ways. A straightforward self-navigation strategy leverages the repeated sampling of the k-space center $k_{x}=k_{y}=k_{z}=0$, from which a self-gating signal can be directly derived (Paul et al., 2015; Lin et al., 2008). Another strategy organizes the $k_{x}=k_{y}=0$ samples across partitions and applies a 1D Fourier transform along $k_{z}$ to generate a stack-wise z-projection, from which respiratory motion or temporal basis functions can be estimated using PCA or related subspace methods (Li et al., 2023; Feng, 2023a; Feng et al., 2020; Zhang et al., 2019; Feng et al., 2016). More recently, SOS has been extended to image-domain navigation by converting fixed-angle navigator stacks into coronal 2D projections using a 2D fast-Fourier transform (FFT) followed by multicoil combination (Chen et al., 2024b; Chen et al., 2024a; Feng, 2023b), enabling more robust tracking of respiratory motion. Collectively, these studies show that SOS data encode motion information at multiple levels, which makes SOS a versatile acquisition strategy for time-resolved MRI.

### 3.4 The DREME-GSMR method

#### 3.4.1 Overview of DREME-GSMR and dual-task learning

**Figure 1.**
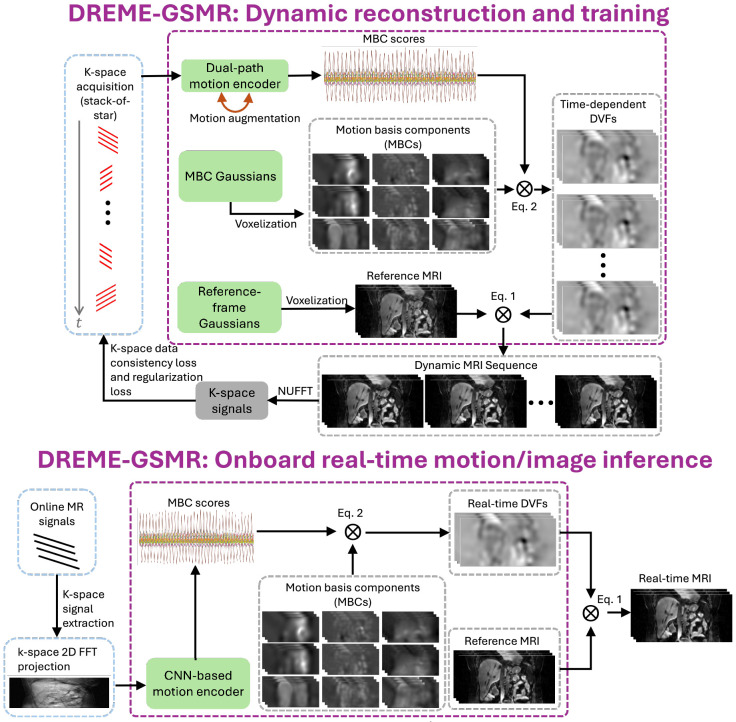
Overview of DREME-GSMR. In the *training stage*, DREME-GSMR simultaneously reconstructs a dynamic MRI sequence and trains a dual-path motion encoder for real-time motion estimation, all based on a pre-treatment MR scan. After training, a motion-compensated, reference-frame MRI is solved along with the MBCs and the motion encoder capable of estimating corresponding MBC scores to represent the dynamic CBCT sequence. In the *onboard real-time motion management stage*, the reconstructed reference MRI, respiratory MBCs, and the CNN-based motion encoder are used to derive real-time MRI, using online MR signals.

Figure 1 illustrates the workflow of the DREME-GSMR framework, which comprises three primary components: reference-frame MRI Gaussians, MBC Gaussians, and a dual path MLP/CNN-based motion encoder, which are responsible for representing the reference-frame anatomy $I_{\mathrm{ref}}$, MBCs $e_{i}(x)$, and MBC scores $w_{i}(t)$, respectively. DREME-GSMR is trained in a ‘one-shot’ fashion, which does not require additional priors, using only the k-space acquisition s(k,t) of a pre-treatment MR scan to generate the time-resolved dynamic sequence of volumetric MR images I(x,t). The dual-task learning is guided by data consistency and regularization losses as defined in Eq. 3. For the data consistency loss, the estimated k-space values at the sampled locations are computed using NUFFT and compared against the acquired k-space measurements. After the dual-task learning, DREME-GSMR yields the up-to-date reference-frame MRI $I_{\mathrm{ref}}$, a patient-specific motion model (i.e., MBCs $e_{i}(x)$ and k-space signal-dependent MBC scores $w_{i}(t)$), and a CNN-based motion encoder that can infer MBC scores based on multi-coil MR signals. For real-time imaging, DREME-GSMR takes the continuously acquired online MR signals to estimate the real-time DVF d(x,t) by the motion model, which is subsequently applied to the reference anatomy to derive the real-time MRI. For real-time target localization, tracking targets (e.g. tumor) can be contoured in the reference-frame MRI $I_{\mathrm{ref}}$ to obtain a target mask, and then the real-time DVF d(x,t) is applied to the target mask to achieve markerless target localization.

The reference-frame MRI Gaussians reconstruct the image $I_{\mathrm{ref}}$ by optimizing the parameters of Gaussian distributions. Because raw MR data are intrinsically complex-valued, with independent real and imaginary components captured by the in-phase and quadrature channels, we instantiate one complex-valued signed 3D Gaussians (Eq. 6) to represent it. The complex-valued Gaussian distribution is voxelized into a dense 3D grid with the CUDA-based Gaussian voxelizer (Eq. 7) to form a complex-valued representation of the MRI volume in voxel space. As the 3D voxelizer is differentiable (Zha et al., 2024), the reference-frame CBCT Gaussians can be iteratively updated using gradients from the data-consistency and regularization losses defined in Eq. 3, thereby achieving the optimization goal.

The MBCs $e_{i}(x)$ of the motion model is represented using MBC Gaussians to allow a sparse representation. Specifically, three spatial levels (i=1, 2, 3) are employed for $e_{i}(x)$ along each Cartesian direction (x, y, z), resulting in a total of nine MBC volumes. For each spatial level, the number of Gaussian points used follows a multi-resolution scheme, to capture coarse-to-fine motion. Following Eq. 5, MBC Gaussians are voxelized and scaled by their corresponding MBC scores to derive the time-dependent motion fields.

The dual-path motion encoder is designed to capture coarse-to-fine motion and enable motion augmentation training to allow robust real-time motion estimation beyond the training distribution. The MLP branch takes the acquired multi-coil k-space signals s(k,t) as input, while the CNN branch takes the 2D FFT projections of the stack-wise k-space signals as input to estimate the MBC scores $w_{i}(t)$. The MLP branch captures global bulk motion while the CNN branch focuses on finer motion and enables detailed motion augmentation. For the CNN-based motion encoder, prior work has shown that training with motion-augmented simulated data enables extrapolation beyond the motion range represented in the original dynamic sequence (Shao et al., 2025a). This capability is particularly important for real-time imaging, where motion patterns encountered during testing may differ substantially from those observed during training.

#### 3.4.2 Motion augmentation training

**Figure 2.**
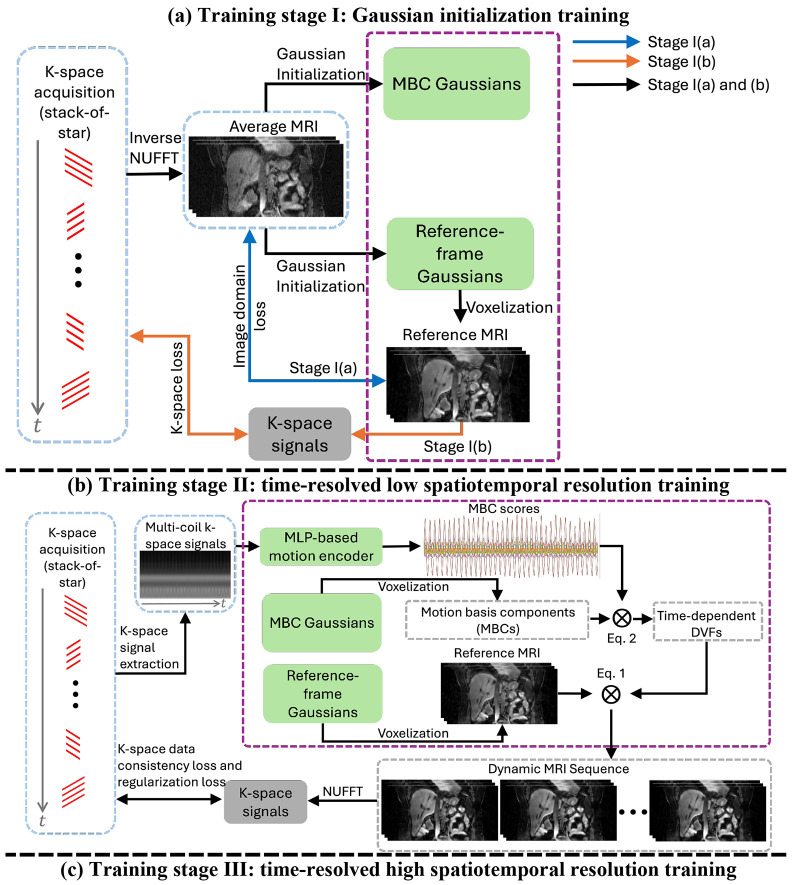

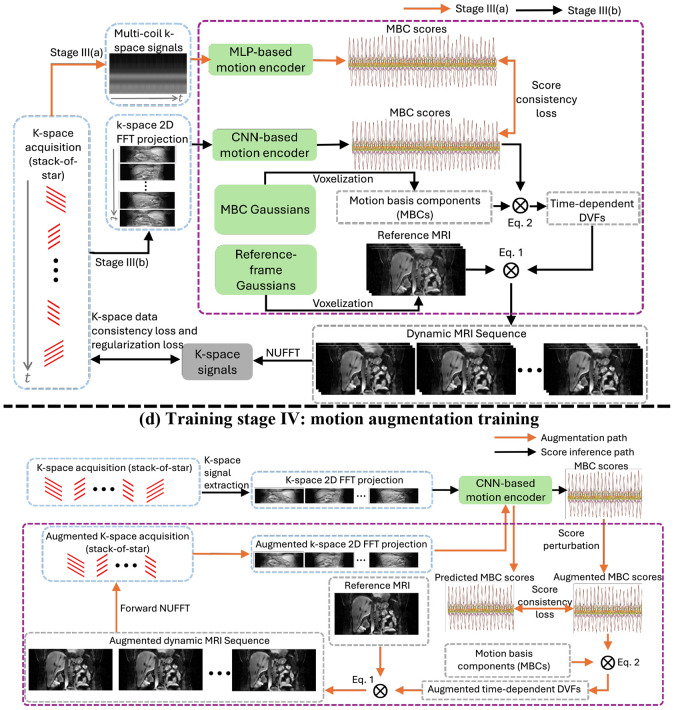
Overview of staged training strategy. (a) Given a sequence of raw k-space signals, a motion-averaged MRI $I_{\mathrm{avg}}$ is reconstructed via inverse NUFFT and sampled for the initialization of the reference-frame MRI Gaussians and the MBCs Gaussians. We used complex-valued 3D Gaussians for the reference-frame MRI and real-valued 3D Gaussians for the MBCs. The positions and intensities of the sampled points are used to position and initialize densities of the Gaussian kernels. In training Stage I(a), the reference-frame MRI Gaussians are trained to reconstruct a motion-averaged MRI with no motion model considered, leveraging a CUDA-based Gaussian voxelizer to decode Gaussians into a reference-frame MRI image $I_{\mathrm{ref}}$ through a L1 loss with the motion-averaged MRI $I_{\mathrm{avg}}$ in the image domain. In training Stage I(b), $I_{\mathrm{ref}}$ is converted to k-space, and the similarity loss is changed to a k-space data consistency loss based on all the k-space data. (b) In Stage II, the reference-frame MRI Gaussians are voxelized and then deformed into a sequence of dynamic MRIs using DVFs generated by summing up the product of MBCs and the corresponding MBC scores. The MBCs are obtained by voxelizing the MBC Gaussians, while the MBC coefficients are derived by an MLP-based motion encoder based on the multi-coil k-space signals. All components are concurrently optimized in Stage II with reduced spatial and temporal resolutions, driven by k-space data consistency and regularization losses. (c) In Stage III(a), a CNN motion encoder is initialized from MBC coefficients predicted by the frozen MLP encoder. In Stage III(b), it is co-optimized with other components at high spatiotemporal resolutions. (d) After Stage III, the reference-frame Gaussians and MBC Gaussians are frozen. The CNN motion encoder’s predicted MBC coefficients are randomly perturbed and combined with frozen MBCs to synthesize augmented DVFs, which warp the reference MRI to generate images with unseen motion. These augmented images are forward-projected to k-space via NUFFT and re-encoded by the CNN. The loss between predicted and augmented MBC coefficients enables the CNN to generalize to a broader motion range for robust real-time estimation.

As shown in Fig. 2(d), motion augmentation was introduced to improve the robustness of the motion encoder to motion states not represented in the original pre-treatment acquisition. Specifically, the solved MBC scores $w_{i}(t)$ were randomly perturbed and applied to the corresponding motion basis components to generate augmented DVFs, which were then used to deform the reference volume and produce dynamic volumes with unseen motion patterns. The augmented volumes were subsequently processed with NUFFT to simulate raw k-space data, from which 2D FFT-based projection images were generated as inputs to the CNN-based motion encoder. The encoder was then trained to predict the perturbed MBC scores, and the predicted scores were compared with the target perturbed coefficients using a similarity loss. The perturbation of the MBC scores was defined as:

(8) $${w^{\prime }}_{i}(t)=r_{1}(t)\times r_{2}(i,k)\times w_{i}(t),$$

where $r_{1}(t)$ acted as a global scaling factor shared across all motion levels i and Cartesian directions k, thereby preserving inter-coefficient correlation within each motion instance, whereas $r_{2}(i,k)$ introduced additional level and direction-specific variations to increase motion diversity. During this stage, the reference image and MBC Gaussians were fixed, and only the CNN-based motion encoder was trained.

#### 3.4.3 Dual-path motion encoder

In our dual-path design, the MLP path captures global bulk motion based on the k-space center, while the CNN path models finer dynamics from the k-space data and enables motion augmentation for robust real-time motion and image estimation.

The MLP-based motion encoder was designed to directly take the acquired multi-coil k-space signals s(k,t) as input to estimate the MBC scores $w_{i}(t)$. Because each coil in a phased-array acquisition has a distinct spatial sensitivity profile, the corresponding k-space signals inherently encode localized anatomical motion. In the SOS trajectory, every readout crosses the in-plane k-space center, such that motion-sensitive samples can be extracted at the central readout position ($k_{x}=k_{y}=0$) across several near-central $k_{z}(k_{z}∼0)$ partitions. Accordingly, for each stack, the complex-valued samples from all coils at the in-plane k-space center of selected central $k_{z}$ locations ($|k_{z}|\leq |k_{\max}|$) were extracted, and their real and imaginary components were concatenated to form the input to the MLP encoder. For example, when the central three partitions were used, the selected samples corresponded to complex signals (separated as real and imaginary values) at $k_{z}=-1,0,1$ and $k_{x}=k_{y}=0$, for each receiver coil. The MLP-based motion encoder has 9 MLP networks, corresponding to the three-level respiratory MBCs $e_{i}(x)$ (with three cartesian components each). All MLPs share an identical architecture composed of three fully connected layers. The first two layers are followed by rectified linear unit (ReLU) activations, while the final layer remains linear to yield a scalar output corresponding to a specific motion component.

The CNN-based motion encoder was designed to enable image-domain motion estimation. For each SOS stack, a central k-space patch of size 3 × 6 was retained, while the remaining k-space samples were zero-filled. A 2D inverse FFT was then applied coil-wise, and the resulting coil images were combined using root-sum-of-squares to generate a single-channel projection image for motion estimation. By restricting the input to the central 3 × 6 k-space patch for each stack, the encoder was encouraged to focus on dominant smooth motion information while reducing sensitivity to fine-scale high-frequency image details, thereby avoiding model overfitting, consistent with prior strategies that derive motion surrogates from the central k-space region (Ahmed et al., 2020; Jiang et al., 2018). The CNN encoder comprised six convolutional blocks with kernel sizes of 7 × 7, 7 × 7, 5 × 5, 5 × 5, 3 × 3, and 3 × 3, and channel numbers of 2, 4, 8, 16, 32, and 32, respectively. Each block consisted of a stride-2 convolution, followed by batch normalization and ReLU activation. The final feature maps were globally averaged and passed through a fully connected layer to predict 9 MBC scores, corresponding to the motion-basis coefficients along different spatial directions and basis levels.

#### 3.4.4 Regularization loss functions

To ensure physiologically plausible motion modeling, several regularization losses were incorporated during the training of DREME-GSMR. The first is a total variation (TV) loss, which mitigates high-frequency noise while preserving anatomical structures and edge integrity:

(9) $$L_{TV}=\frac{1}{N_{voxel}}\sum_{i}|\nabla I_{\text{ref}}(x_{i})|$$

where $N_{\mathrm{voxel}}$ denotes the number of voxels, i denotes the voxel index, and ∇ denotes the gradient operator.

The other four loss functions were introduced to regularize the respiratory motion model. Firstly, to resolve ambiguities in the spatiotemporal decomposition (Eq. 2) of the low-rank motion model, we incorporate a normalization loss to promote the normality of MBCs $e_{i}(x)$:

(10) $$L_{MBC}=\frac{1}{9}\sum_{k=x,y,z}\sum_{i=1}^{3}\left({|{\left\|e_{i,k}\right\|}_{2}^{2}-1|}^{2}\right),$$

where ${\left\|\cdot \right\|}_{2}$ is the L2 norm. Secondly, a zero-mean regularization is applied to the MBC scores $w_{i}(t)$:


(11) $$L_{ZMS}=\frac{1}{9}\sum_{k=x,y,z}\sum_{i=1}^{3}{|\frac{1}{N_{t}}\sum_{t}w_{i,k}(t)|}^{2},$$

This loss penalizes any time-independent baseline offsets in the MBC scores, effectively placing the mean of the estimated respiratory motion around the origin. Our previous work (Shao et al., 2025a) demonstrated that incorporating this loss improves overall real-time motion estimation accuracy in digital phantom experiments. Thirdly, a Jacobian regularization term was introduced to suppress anatomically implausible organ motion (Chen et al., 2025; Heyde et al., 2016; Chun and Fessler, 2009) by constraining the local deformation induced by the dynamic DVFs:

(12) $$L_{Jac}=\frac{1}{\sum_{t}|\Omega_{t}|}\sum_{t}\sum_{x\in \Omega_{t}}{\left(det\left(J_{\emptyset_{t}}(x)\right)-1\right)}^{2},$$

where $J_{\emptyset_{t}}(x)=\nabla \emptyset_{t}(x)$ represents the Jacobian matrix of the transformation $\emptyset_{t}(x)=x+d(x,t)$ at point x, while $\Omega_{t}$ denote the region of interest (ROI) at frame t. An ROI mask was generated from the reference image using intensity thresholding to separate the low-intensity air/background and focus the Jacobian regularization on the anatomy. This form penalizes large deviations of the determinant of the Jacobian matrix from 1, thereby discouraging unrealistic local compression, expansion, and folding in the estimated deformation field. Finally, a frequency regularization term was incorporated to suppress non-physiological oscillations in the motion scores produced by the motion encoder by penalizing abnormal spectral peaks. Specifically, the temporal scores were transformed into the frequency domain, and the average spectral power in predefined frequency bands was compared with that in neighboring baseline bands:

(13) $$L_{Freq}=\frac{1}{N_{peak}}\sum |\bar{P}(C)-\bar{P}(B)|,$$

where $\bar{P}(C)$ and $\bar{P}(B)$ denotes the mean spectral power within the target frequency band C and its corresponding neighboring baseline band B, respectively, and $N_{\mathrm{peak}}$ denotes the number of predefined frequency bands. In implementation, the target frequency bands were defined in the temporal sampling domain of the motion-score sequence. Specifically, one score sample was associated with each stack, and each stack spanned $n_{kz}$ spokes with TR of $t_{r}$. Therefore, the score sampling rate was $f_{s}=\frac{1}{t_{r}n_{kz}}$, yielding a Nyquist frequency of $f_{N}=\frac{1}{2t_{r}n_{kz}}$. A predefined scanner-related reference frequency (60.7353 Hz, reflecting acquisition noise), its higher-order harmonics, and their folded aliases were then mapped into this Nyquist range to define the target bands, while adjacent side bands served as local spectral baselines. By discouraging abnormal peaks in the score spectrum, this regularization reduces the tendency of the motion encoder to model acquisition noise or unstable temporal oscillations instead of true anatomical motion.

#### 3.4.5 Training strategy

To initialize Gaussians, a motion-averaged MRI $I_{\mathrm{avg}}(x)$ is reconstructed via inverse NUFFT using coil-compressed k-space data of all radial spokes. For reference-frame MRI Gaussians, M points are sampled uniformly from $I_{\mathrm{avg}}(x)$, preserving their corresponding positions and densities. For MBC Gaussians, $M_{1}$, $M_{2}$, and $M_{3}$ points are sampled using gridbased sampling for three spatial levels to represent coarse-to-fine ($M_{3}>M_{2}>M_{1}$) motion. With the negative-permitting voxelizer, we used one complex-valued Gaussian cloud to represent the reference-frame MRI, and 9 (3 × 3) real-valued Gaussian clouds to represent the MBCs, one Gaussian cloud for each spatial level and Cartesian direction.

As shown in Fig. 2, DREME-GSMR follows a progressive four-stage training strategy after initialization to improve learning stability and avoid convergence to local optima while solving the dynamic sequence of MRIs, consistent with prior dynamic (Xie et al., 2025; Shao et al., 2024) and real-time motion estimation (Shao et al., 2025b; Shao et al., 2025a) frameworks.

In Stage I (Fig. 2(a)), we train the reference-frame Gaussians (Eq. 6) to reconstruct the motion-averaged MRI with no anatomical motion considered. It is further divided into two steps. In Stage I(a), the loss function is defined in the image domain between the motion-averaged MRI $I_{\mathrm{avg}}(x)$ and the estimated reference-frame MRI $I_{\mathrm{ref}}(x)$:

(14) $$L_{I}^{a}=\frac{1}{N_{voxel}}\sum_{x}{\left\|I_{ref}(x)-I_{avg}(x)\right\|}_{1},$$

Since $I_{\mathrm{avg}}(x)$ is reconstructed from coil-compressed k-space data, it exhibits artifacts arising from coil compression and anatomical motion. In Stage I(b), we aim to suppress artifacts related to coil compression that were introduced into $I_{\mathrm{ref}}(x)$ during Stage I(a) training, such that the remaining artifacts primarily reflect anatomical motion. To achieve this, the loss function is reformulated as a k-space data consistency loss computed over all acquired k-space samples, combined with the TV regularization loss (Eq. 7):

(15) $$L_{I}^{b}=E[{\left\|ℱ[I_{ref}(x)]-s(k)\right\|}_{1}]+\lambda_{TV}L_{TV},$$

where $\lambda_{\mathrm{TV}}$ denotes the weighting factor of the TV loss. $I_{\mathrm{ref}}$ is obtained via voxelizing the reference-frame Gaussians and is subsequently converted to k-space via NUFFT for loss calculation, with no motion considered.

In Stage II (Fig. 2(b)), we train the reference-frame Gaussians (Eq. 6), the MBC Gaussians (Eq. 4), and the MLP motion encoder (Sec. 3.4.3) simultaneously with the k-space data consistency loss and the regularization losses (Eqs 9-12) to learn bulk motion with a low temporal resolution:

(16) $$L_{II}=E[{\left\|ℱ[I_{ref}(x)]-s(k)\right\|}_{1}]+\lambda_{TV}L_{TV}+\lambda_{MBC}L_{MBC}+\lambda_{ZMS}L_{ZMS}+\lambda_{Jac}L_{Jac},$$

where $\lambda_{\mathrm{MBC}}$, $\lambda_{\mathrm{ZMS}}$, and $\lambda_{\mathrm{Jac}}$ denote the weighting factors of the MBC loss, the zero-mean score loss, and the Jacobian loss, respectively. Specifically, MBCs $e_{i}(x)$ (voxelized from MBC Gaussians) and MBC scores $w_{i}(t)$ estimated from the MLP motion encoder are combined via Eq. 2 to obtain dynamic DVFs d(x,t). By warping the reference-frame MRI using the DVFs, time-resolved dynamic MRIs are obtained and then converted to k-space to compare with the acquired k-space. In this stage, the in-plane image resolution was downsampled by a factor of two, while the through-plane resolution was preserved. In addition, for each stack, its two adjacent temporal neighbors were combined on-the-fly, resulting in a reduced effective temporal resolution. This coarse spatiotemporal training strategy emphasizes dominant bulk-motion patterns while suppressing high-frequency fluctuations and noise during early optimization. As a result, the model can first establish a stable low-resolution motion representation, which improves training robustness and reduces the risk of convergence to poor local minima.

In Stage III (Fig. 2 (c)), the CNN motion encoder (Sec. 3.4.3) was incorporated and trained together with the reference-frame Gaussians (Eq. 6) and the MBC Gaussians (Eq. 4) to recover motion patterns at high spatiotemporal resolutions. At the beginning of this stage, Stage III(a) first initialized the CNN encoder by matching its motion scores to those predicted by the MLP motion encoder, which was trained in Stage II and frozen in Stage III. The initialization objective was defined as:

(17) $$L_{III}^{a}=MAE(Score_{MLP}-Score_{CNN}).$$

Following this warm start, Stage III(b) refined the full model using the native MRI spatial resolution and stack-wise temporal resolution with the objective:

(18) $$L_{III}^{b}=E[{\left\|ℱ[I_{ref}(x)]-s(k)\right\|}_{1}]+\lambda_{TV}L_{TV}+\lambda_{MBC}L_{MBC}+\lambda_{ZMS}L_{ZMS}+\lambda_{Jac}L_{Jac}\;\;+\lambda_{Freq}L_{Freq},$$

where $\lambda_{\mathrm{Freq}}$ is the weighting factor for the frequency regularization term (Eq. 13). Compared with Stage II, this stage restores the original spatial and temporal resolutions of the acquisition, allowing the model to capture finer and more rapidly varying motion dynamics. Meanwhile, the frequency regularization constrains abnormal oscillations in the CNN-derived motion scores, reducing the influence of acquisition-related noise and improving the fidelity of the recovered motion representation.

Finally, Stage IV served as a motion augmentation stage (Fig. 2 (d)), in which the CNN-based motion encoder was fine-tuned using augmented motion patterns that were not observed during the training stages. The objective was defined as the mean absolute error between the augmented MBC scores and the scores predicted by the CNN motion encoder:

(19) $$L_{IV}=MAE(Score_{Aug}-Score_{CNN}).$$

This stage was designed to improve the generalizability of the motion encoder to previously unseen motion patterns, thereby enhancing its robustness for real-time motion estimation.

## 4. Experiments

### 4.1 Evaluation datasets and metrics

We evaluated DREME-GSMR using the Extended Cardiac Torso (XCAT) digital phantom (Segars et al., 2010) simulations, physical phantom measurements, and clinical data. Both physical phantom and clinical data are acquired with the stack-of-stars trajectory from a 1.5T MR-LINAC. The XCAT simulation study provided ‘ground-truth’ anatomy and motion, enabling quantitative evaluation of both image reconstruction quality and motion estimation accuracy. The physical phantom experiments offered known motion trajectories under realistic acquisition conditions. Finally, the clinical dataset facilitated an assessment of DREME-GSMR’s clinical potential and applicability.

#### XCAT simulation study.

To test DREME-MR’s capacity for one-shot learning of irregular respiratory motion, we generated six XCAT motion scenarios that differed in frequency, amplitude, and baseline behavior. Scenario X1 modeled regular breathing with a 20 mm amplitude in superior-inferior (SI) direction and a 10 mm amplitude in anterior-posterior (AP) direction; X2 introduced a sudden baseline shift in the middle of the scan; X3 combined amplitude variations with a slow baseline drift; X4 featured a gradual reduction in breathing frequency with increasing amplitude; X5 simulated a slow breathing scenario; and X6 simultaneously varied baseline, amplitude, and frequency. A 30 mm-diameter liver tumor was inserted into the upper liver segment to serve as the tracking target. Because XCAT produces real-valued MR data, random phase angles were assigned to organs and tissues to simulate complex signals. Each motion scenario yielded 673 dynamic MR frames, equivalent to a 4.75-min scan with a 423 ms temporal resolution per SOS stack.

To match realistic MR acquisition, we simulated the corresponding multi-coil k-space data after generating the ‘ground-truth’ XCAT dynamic MRI volumes. A steady-state spoiled gradient echo sequence with a repetition time (TR) of 4.5 ms was used. For each dynamic volume, sampled at a temporal resolution of 423 ms, 94 spokes per stack were simulated in k-space following SOS golden-angle radial sampling, with each spoke containing 368 readout points along each radial spoke with 8 receiver coils. Images were reconstructed with a volume size of 368 × 368 × 94 at a 2 × 2 × 3 *mm*^3^ voxel resolution.

The performance of two learning tasks was separately evaluated. For dynamic MRI reconstruction (learning task 1), DREME-GSMR was trained separately on the six motion scenarios (X1-X6), and the image quality of the reconstructed dynamic MRI and the accuracy of the resolved respiratory motion were evaluated on the same motion scenarios. For real-time imaging (learning task 2), the model trained on a given motion scenario (e.g., X1) was cross-tested on the remaining scenarios (X2-X6) to assess its generalizability to unseen respiratory motion patterns.

In the digital phantom study, where ground-truth volumetric images were available, both the reconstructed dynamic MRIs and the estimated real-time MRIs were quantitatively evaluated. The image quality was evaluated using the structural similarity index measure (SSIM) (Zhou et al., 2004). The motion estimation accuracy was evaluated by the center-of-mass error (COME) and the Dice similarity coefficient (DSC) of tracked tumor contours. The COME is defined as the Euclidean distance between the estimated and ‘ground-truth’ centers of mass of the target. The DSC measures the spatial overlap between the estimated and ‘ground-truth’ contours. Specifically, we contoured lung tumors from reference-frame MRI images of each motion scenario and then propagated the tracking masks to the dynamic/real-time MRI instances using the DVFs solved by DREME-GSMR. These propagated contours were compared with the ‘ground-truth’ tumor contours using the COME and DICE metrics to quantify motion estimation accuracy.

#### Physical phantom study.

A physical phantom study was conducted using a QUASAR MRI^4D^ Motion Phantom, which consists of a water-filled outer body and a movable cylindrical insert and supports programmable motion along the SI direction. The imaging insert comprised an inner cylinder filled with a CuSO_4_ solution and an air-filled spherical target mounted within the cylinder. Under T1-weighted imaging, the inner cylinder appeared bright while the spherical target and outer body were dark, providing clear contrast for motion evaluation. The inner cylinder was programmed to move along the SI direction in two irregular and two regular motion patterns. The two irregular motions (Motion 1 and Motion 2) had mean peak-to-valley displacements of 22.15 ± 2.32 mm over 95 cycles and 20.04 ± 4.59 mm over 47 cycles, respectively. The two regular motions (Motion 3 and Motion 4) were sinusoidal, with a period of 4 s and an amplitude of 30 mm, and a period of 3 s and an amplitude of 24 mm, respectively. Data were acquired on a 1.5T Elekta Unity MR-LINAC (Elekta AB, Stockholm, Sweden) at the UT Southwestern Medical Center with a TR of 4.5 ms and 8 receive coils. A total of 673 stacks were continuously acquired using SOS golden-angle radial sampling for ~5 minutes. The reconstructed images had a spatial resolution of 2 × 2 × 3 *mm*^3^ with a matrix size of 368 × 368 × 94.

Similar to the XCAT study, for dynamic MRI reconstruction, DREME-GSMR was trained separately for each of the four motion scenarios, and the accuracy of the solved motion was evaluated within the corresponding scenario. For real-time imaging, a cross-validation scheme was used to assess motion estimation accuracy. Specifically, the spherical target inside the phantom was contoured, and its motion was compared with the programmed ‘ground-truth’ curves through the COME metric.

#### Clinical study.

Clinical data were acquired on the same 1.5T MR-LINAC as the physical phantom study, from 6 healthy volunteers and 20 patients. The volunteers were imaged under free-breathing conditions, whereas the patient cases were acquired under their corresponding treatment-specific motion-management protocols (free-breathing with or without abdominal compression). T1-weighted images were obtained with a reconstructed matrix size of approximately 500 × 500 × 61 – 79 and a spatial resolution of 1.5 × 1.5 × 3 *mm*^3^, acquired with a TR of 5.1 ms and 8 receive coils. A total of 878-921 radial stacks were continuously acquired for each scan, corresponding to a temporal resolution of ~400 ms per stack and a scan time of ~6 mins.

For clinical data, where no absolute ‘ground truth’ exists, each subject had two sequential scans (A/B) to enable cross-scan validation: the model trained on scan A was used to infer real-time motion/imaging on scan B, and evaluated against the dynamic reconstruction results of a reference model trained on scan B (and vice versa). For each case, the liver was contoured, and its COME was calculated between the real-time prediction and the corresponding ‘pseudo-ground-truth’ reconstruction. This evaluation strategy was designed to assess the generalizability of the proposed framework in a realistic clinical setting, where a pre-treatment scan is available for model training, whereas real-time inference must be performed on online MR signals acquired under potentially different motion patterns.

### 4.2 Implementation details

Implementation details of DREME-GSMR are as follows: (a) We implemented DREME-GSMR in PyTorch 1.13.1(Paszke et al., 2019), and trained with the Adam optimizer (Kingma and Ba, 2014); (b) The NUFFT operator was adopted from the TorchKbNufft library (Muckley et al.); (c) For the XCAT and clinical studies, readouts at the three $k_{x}=k_{y}=k_{z}=0$ central locations around $k_{z}=0$ were used as input for the MLP-based motion encoder. In the physical phantom study, nine central $k_{z}$ locations were used to better capture the locally-restricted motion of the phantom. (d) Due to the wide variations of raw k-space signals s(k,t) across different coils, z-score normalization was applied to the real and imaginary channels before inputting them into the motion encoder. (e) Regarding DREME-GSMR’s Gaussian initializations, we set the number of cloud points as M=100,000 to initialize reference-frame Gaussians, and for the three levels of MBC Gaussians, we set $M_{1}=15^{3}$, $M_{2}=20^{3}$, and $M_{3}=25^{3}$, respectively. (f) Weighting factors were set to $\lambda_{\mathrm{TV}}=2\times 10^{-6}$, $\lambda_{\mathrm{MBC}}=1\times 10^{-2}$, $\lambda_{\mathrm{ZMS}}=1\times 10^{-4}$, $\lambda_{\mathrm{Jac}}=1\times 10^{-1}$, and $\lambda_{\mathrm{Freq}}=1$. (g) The numbers of epochs for Stage I(a), Stage I(b), Stage II, Stage III(a), Stage III(b), and the motion augmentation stage were 400, 300, 1200, 5000, 1500, and 1000, respectively. (h) The learning rate of the MLP motion encoders was set to 2 × 10^−3^ and 5 × 10^−4^ for the physical phantom data, and the clinical/XCAT datasets, respectively. The learning rate of the CNN was set to 2 × 10^−3^ at Stage III(a) and changed to 1 × 10^−4^ for later stages. (i) The motion augmentation factors were set to $r_{1}\in [0.2,2.6]$ and $r_{2}\in [0.4,1.6]$. For the physical phantom study, in which motion occurred only along the SI direction, motion augmentation was applied only along that direction.

### 4.3 Comparison and ablation studies

#### 4.3.1 Comparison methods

We compared DREME-GSMR with XD-GRASP (Feng et al., 2016), a PCA-based motion modeling method, and the DREME-MR model (Shao et al., 2025b), which is considered the state-of-the-art.

As a reference method for SOS 4D-MRI, we used XD-GRASP, an established respiratory motion-resolved reconstruction framework. However, it reconstructs retrospectively sorted respiratory states rather than continuous real-time volumes. It was implemented with 10 respiratory phases, which can limit the representation of irregular or nonperiodic motion. We therefore used XD-GRASP to highlight the advantage of dynamic MRI in capturing motion variations that cannot be fully described by phase-binned 4D-MRI.

To our knowledge, we did not identify a publicly available framework that jointly performs patient-specific dynamic MRI reconstruction and real-time volumetric MRI inference within a single unified pipeline. We therefore compared DREME-GSMR with a PCA-based motion modeling baseline that enables dynamic reconstruction and real-time imaging. Specifically, by sorting a pre-treatment MRI into 10 respiratory phases (amplitude-based sorting using XD-GRASP), we derived a patient-specific motion model by applying PCA to the resulting 4D-MRI. Using the end-of-exhale phase as the reference phase, PCA motion bases were obtained from the inter-phase DVFs of the pre-treatment 4D-MRI. With the known, frozen PCA motion bases, we trained a variant of DREME-GSMR by updating an MLP-based motion encoder to estimate the PCA motion coefficients directly from the MR signals while simultaneously updating the reference-phase image, generating a dynamic MRI set from the same pretreatment MRI scans. Conceptually, this baseline is similar to STINR-MR (Shao et al., 2024) with modifications that enable real-time inference. The key difference from DREME-GSMR is that the motion bases in the PCA-based modeling approach are predefined from PCA, whereas those in DREME-GSMR are learned and optimized jointly within the reconstruction framework. In addition, DREME-GSMR used a dual-path MLP/CNN motion encoder, with additional motion augmentation performed. In contrast, the PCA motion modeling approach only used an MLP motion encoder without additional motion augmentation.

We also compared DREME-GSMR with the state-of-the-art DREME-MR model. For DREME-MR, the network architecture and settings were kept the same as originally reported, except that we excluded components that are responsible for cardiac motion (Shao et al., 2025b) and enabled multi-resolution training (Sec. 3.4.5) for a fair comparison with DREME-GSMR. Specifically, we removed the cardiac MBCs $e_{i}^{c}(x)$, the respiratory frequency loss function $L_{r}$, and reduced the number of MLP networks from 12 to 9 for the motion encoder.

#### 4.3.2 Ablation studies

We conducted two ablation studies to analyze key design choices in DREME-GSMR. First, to evaluate the benefit of the proposed dual-path motion encoder, we compared DREME-GSMR with two single-encoder variants, denoted DREME-GSMR-MLP and DREME-GSMR-CNN, in which only the MLP-based or CNN-based motion encoder was used under the same overall training framework. Notably, for DREME-GSMR-MLP, the same motion augmentation strategy (Fig. 2) was used, except that simulated central k-space signals rather than 2D FFT-based projection images were used as encoder inputs to ensure a fair comparison. Second, to evaluate the impact of data quantity on dynamic reconstruction and real-time imaging performance and to examine the potential of the proposed method under different undersampled conditions, we repeated the experiments using 20%, 25%, 50%, and 75% of the acquired radial stacks, and compared the results with those obtained using the full dataset.

## 5. Results

### 5.1 The XCAT study results

**Figure 3.**
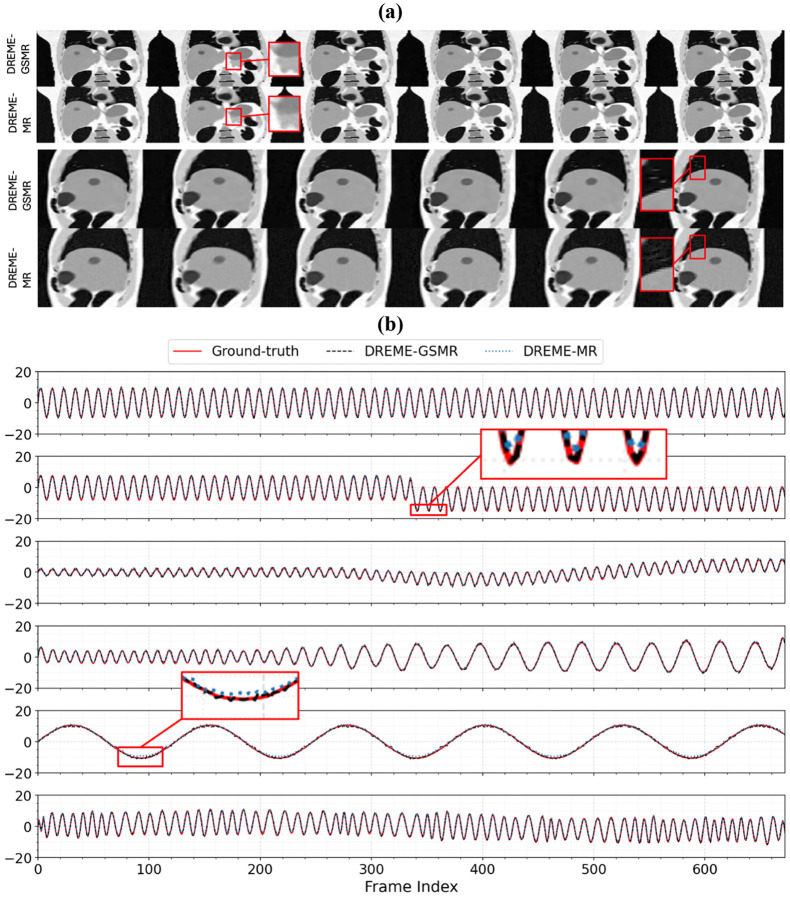

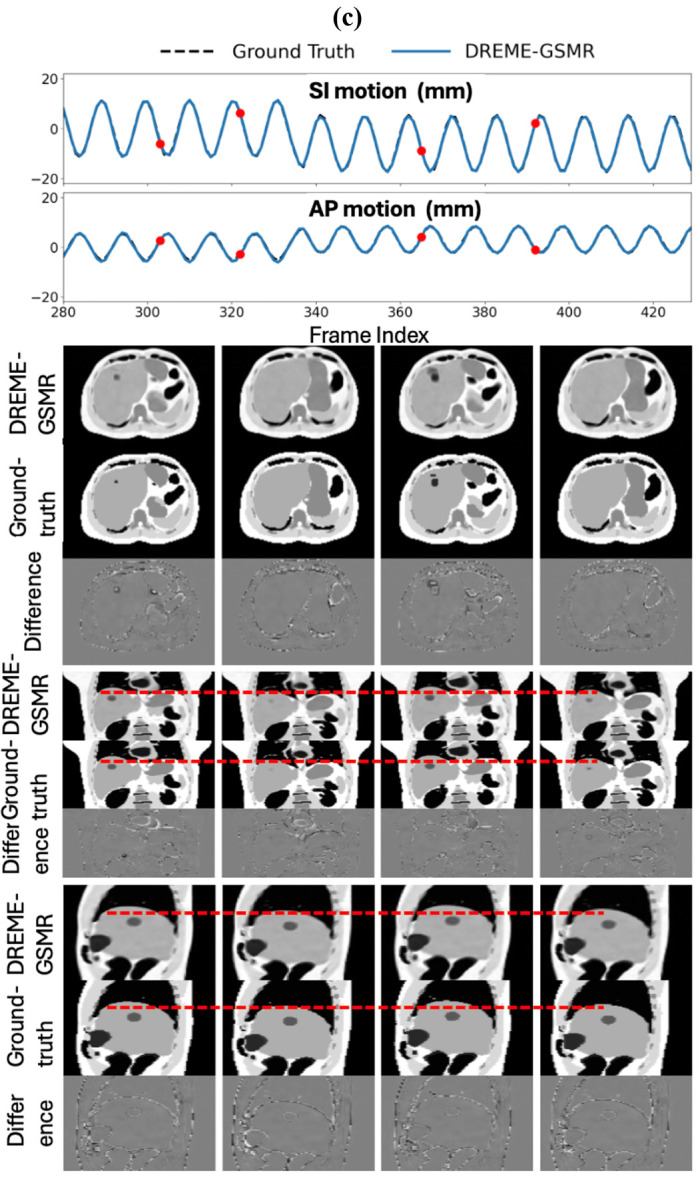
XCAT study results visualizations. (a) Comparison of reconstructed reference-frame MRIs from six motion scenarios (X1-X6, from left to right) between DREME-MR and DREME-GSMR. (b) Liver tumor center-of-mass trajectories in the SI direction of the XCAT study. The DREME-MR and DREME-GSMR were trained on the X1 scenario and tested on all six (X1-X6) scenarios. (c) An example of DREME-GSMR’s dynamic reconstruction results in the XCAT study (training case X2). The first two rows show the estimated motion curves along the SI and AP directions. The following rows compare the reconstructed MR images with the ‘ground-truth’ images at four time points at axial, coronal, and sagittal views. The window widths of the difference images are half of those of the MR images.

Figure 3(a) presents a comparison between reference MRIs reconstructed using DREME-MR and DREME-GSMR across six motion scenarios (X1-X6) in coronal and sagittal views for XCAT. Overall, both models reduced artifacts and motion-induced blurring substantially, providing better-defined anatomical structures. DREME-GSMR shows improved reconstruction image quality over DREME-MR with higher SSIM scores (Table I). The reconstructions obtained with DREME-GSMR exhibit sharper anatomical structures, as highlighted by zoomed-in panels. This arises from DREME-GSMR’s continuous 3D Gaussian representation, which imposes spatial smoothness while allowing high-definition anatomy description. Unlike DREME-MR, which relies on an INR and tends to over-smooth fine anatomical structures due to its inductive low-frequency bias, DREME-GSMR more effectively preserves high-frequency details, resulting in enhanced image fidelity.

Figure 3(b) compares the tumor center-of-mass SI motion trajectories estimated by DREME-MR and DREME-GSMR against the reference XCAT ‘ground truth’. The DREME-MR and DREME-GSMR were trained on the X1 motion scenario with regular breathing cycle/amplitude and tested on all scenarios (X1-X6). Both models faithfully capture the motion trends and achieve sub-voxel tumor tracking accuracy (0.50-0.74 mm) in both dynamic reconstruction and real-time imaging tasks (Table I). However, quantitatively in Table I, DREME-GSMR consistently demonstrates improved image quality with lower COME scores. Overall tracking accuracy of both models remains high, indicating their effectiveness in capturing diverse motion irregularity across a wide range of motion amplitudes (X1-X6) with superior generalizability to unseen motion patterns. Notably, DREME-GSMR achieved comparable or slightly better performance to DREME-MR while requiring 70% of the computation time.

Figure 3(c) shows representative dynamic MRI reconstructions by DREME-GSMR for training case X2 at four time points. The reconstructed SI and AP motion trajectories closely match the ‘ground truth’, and the axial, coronal, and sagittal views demonstrate a good agreement between the reconstructed and ‘ground-truth’ XCAT volumes. In particular, the deformation of the liver and tumor is well preserved, while the residual difference images show only minor errors, primarily around anatomical boundaries.

### 5.2 The physical phantom study results

**Figure 4.**
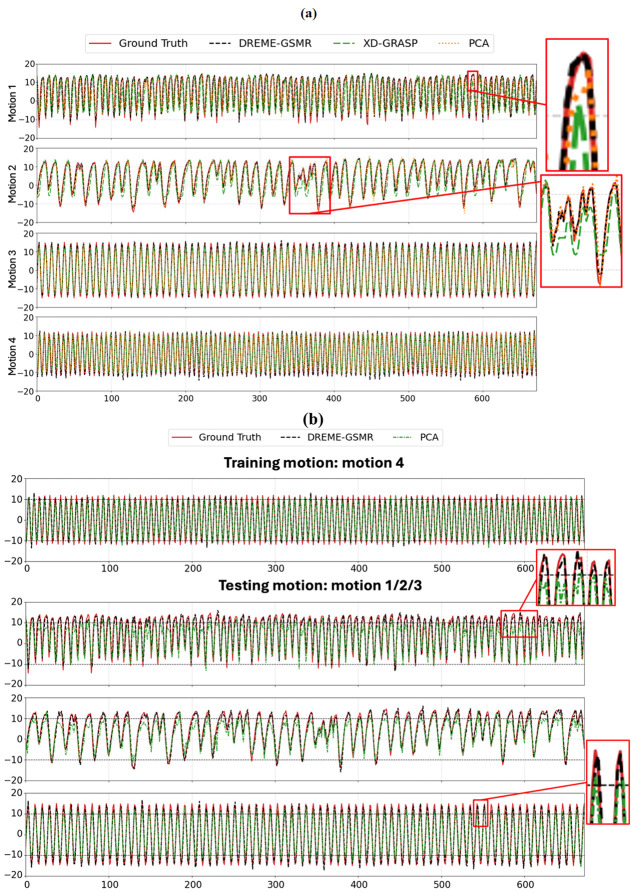

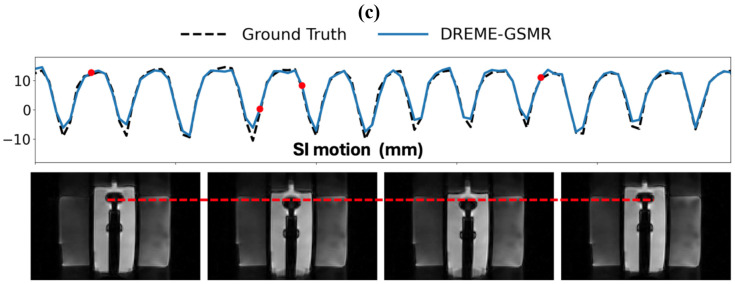
Physical phantom study results visualizations. (a) SI center-of-mass trajectories of the spherical tracking target in the physical phantom study for the dynamic reconstruction of Motions 1-4. Trajectories from DREME-GSMR, XD-GRASP, and PCA reconstructions are overlaid with the programmed ‘ground-truth’ curves. Enlarged views highlight representative regions with visible differences among the methods. (b) Real-time motion estimation trajectories in the physical phantom study. DREME-GSMR and PCA were trained on Motion 4. The top row shows the training motion, and the lower three rows show testing on unseen motions 1-3. The horizontal dashed lines indicate SI positions of −10 and +10 mm. (c) Dynamic MRI images of one phantom case (motion 1) resolved by DREME-GSMR. The top row compares the reconstructed SI motion trajectory with the programmed ‘ground-truth’ curve, and the red markers indicate the four time points displayed below. The second row shows sagittal images at these four time points.

Figure 4(a) compares the SI center-of-mass trajectories of the spherical tracking target obtained from the dynamic reconstructions of the four phantom motion scenarios using DREME-GSMR, XD-GRASP, and PCA. Overall, DREME-GSMR showed the best agreement with the programmed ‘ground-truth’ trajectories across all cases. As summarized in Table II, DREME-GSMR achieved the lowest dynamic reconstruction error in all four experiments, with an average error of ~1.20 mm. In comparison, the average error was ~3.04 mm for XD-GRASP and ~2.50 mm for the PCA-based motion modeling method (PCA). The zoom-in views in Fig. 4(a) further show that DREME-GSMR more faithfully preserved motion amplitude and cycle-to-cycle variations, particularly for the more irregular motion patterns in Motion 2. XD-GRASP exhibited the largest deviations, as its amplitude phase-sorting strategy represents motion using only a limited number of motion states and is therefore less capable of capturing irregular temporal variations, whereas PCA showed intermediate performance.

For real-time imaging, Fig. 4(b) compares DREME-GSMR and PCA when trained on Motion 4 and applied to unseen motions. XD-GRASP was not included because it was not designed for real-time motion estimation. Although PCA achieved moderate tracking accuracy, DREME-GSMR followed the ‘ground-truth’ trajectories more consistently. In the zoomed-in panels, PCA shows clear motion undershooting, whereas DREME-GSMR remains closely aligned with the ‘ground truth’. These observations are consistent with the quantitative results in Table II, where DREME-GSMR achieved lower COME than PCA for all cross-scenario real-time imaging experiments.

Figure 4(c) shows an example of DREME-GSMR-resolved dynamic MRI for Motion 1. The reconstructed SI trajectory closely matches the programmed ‘ground-truth’ motion, and the sagittal images at the four selected time points clearly demonstrate the displacement of the inner cylinder relative to the fixed reference line.

### 5.3 The clinical study results

**Figure A.1.**
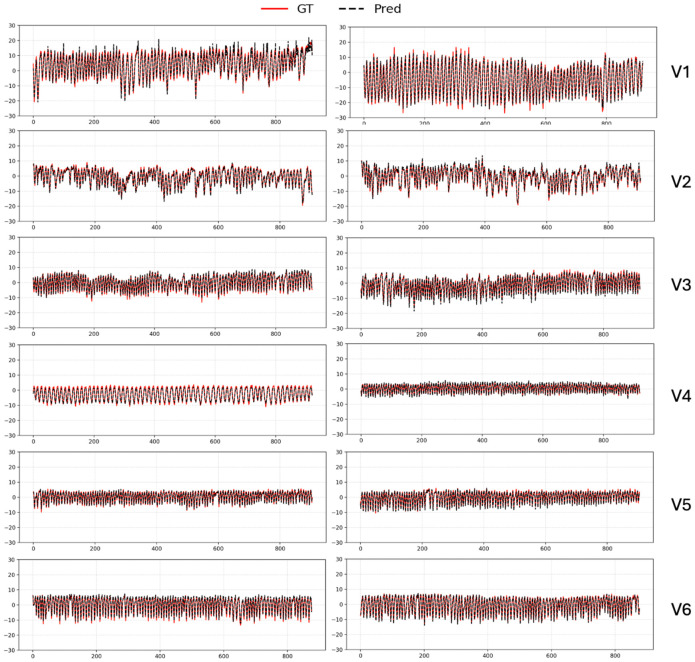

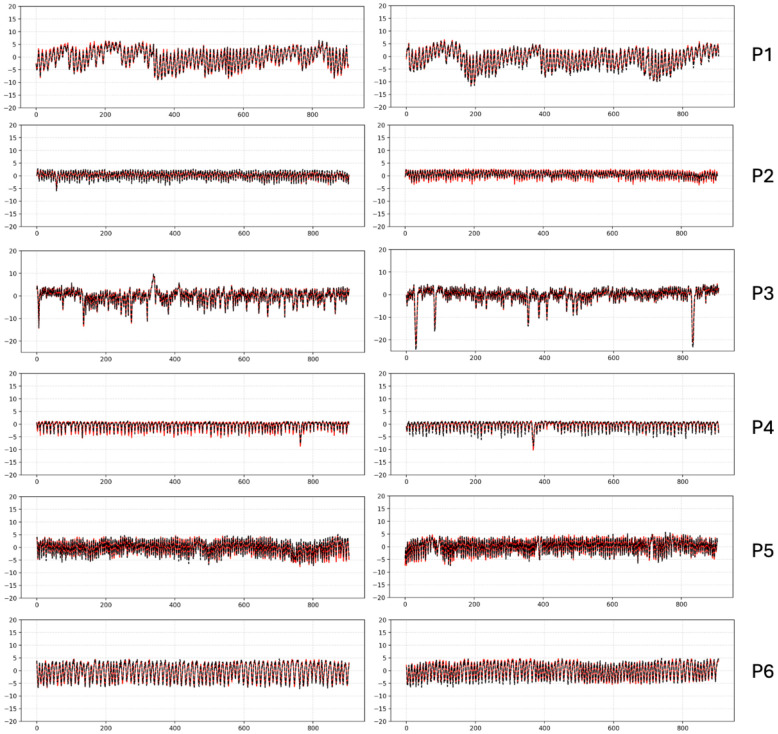

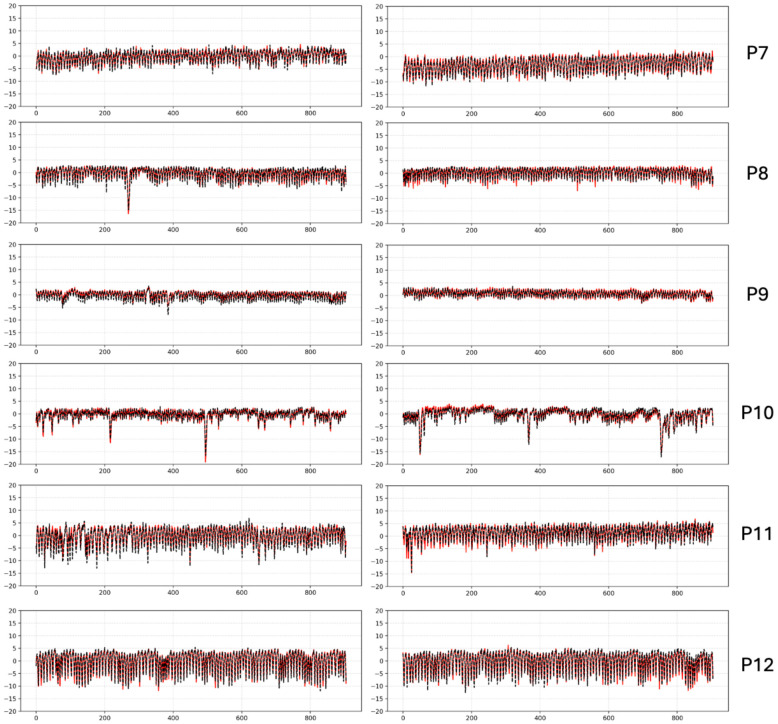

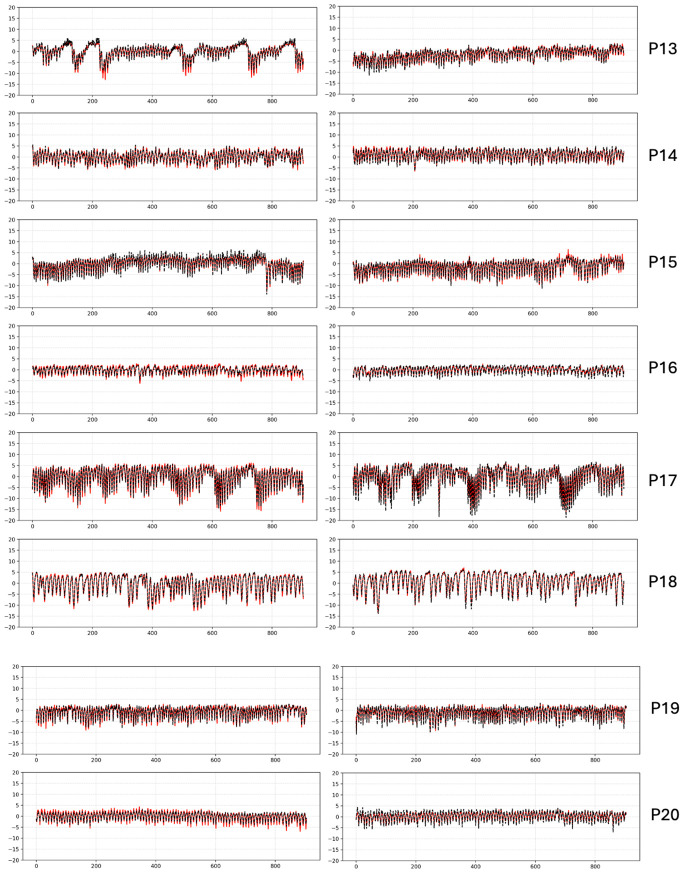
Cross-scan testing results for clinical dataset.

The clinical dataset consisted of two sequential scans from 6 healthy volunteers and 20 patients. The complete cross-scan testing results are provided in Appendix Table A.1 and Fig. A.1. Because the volunteers were imaged under free-breathing conditions, whereas the patients were scanned under treatment-specific motion-management protocols (free-breathing or free-breathing with abdominal compression), the two groups were analyzed separately. As summarized in Table III, the volunteers exhibited substantially larger respiratory motion than the patients, with an average SI peak-to-valley motion range of 10.26 ± 5.61 mm versus 4.47 ± 2.63 mm, respectively. Using the dynamic reconstruction results as pseudo ‘ground truth’, DREME-GSMR achieved a mean 3D liver localization error of 1.31 ± 0.82 mm for the volunteer group and 0.96 ± 0.64 mm for the patient group, indicating that the proposed method maintained good real-time motion estimation accuracy in both free-breathing and motion-managed clinical settings.

**Figure 5.**
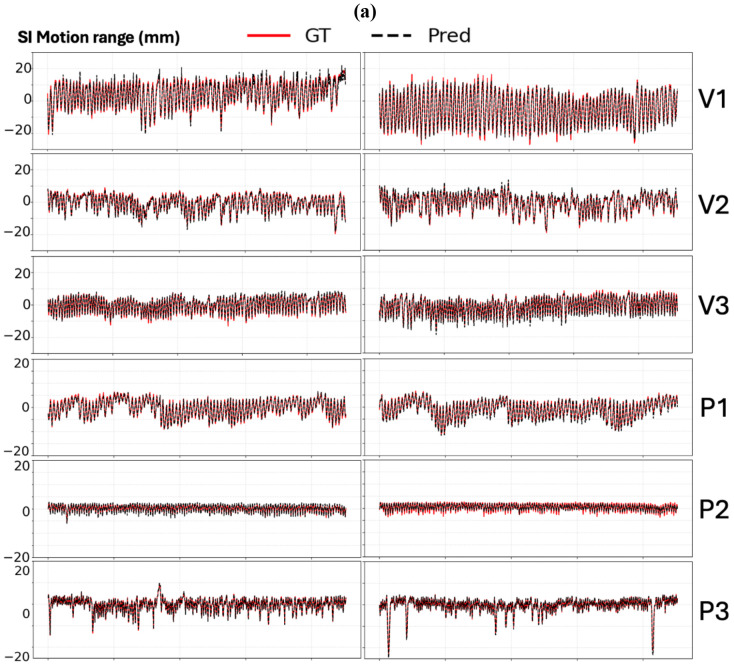

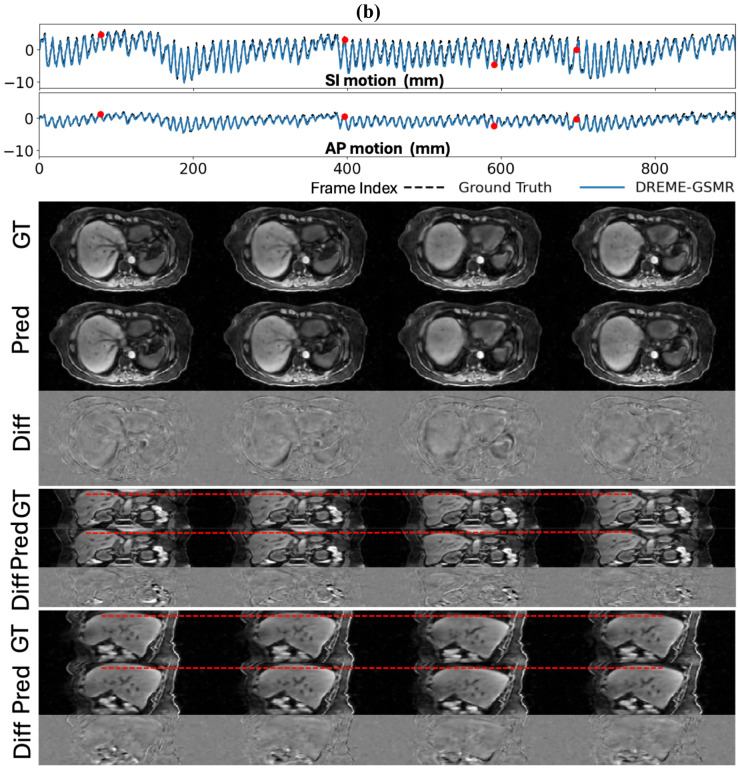
Clinical study result visualizations. (a) Representative liver SI motion trajectories from clinical scans of volunteers (V1-V3) and patients (P1-P3) under cross-scan testing. For each case, the model was trained on one scan and tested on the paired scan, and the dynamic reconstruction of the test scan was used as the pseudo ‘ground truth’. The two columns show the two directional cross-scan testing experiments between the paired A/B scans. (b) Example of reconstructed clinical scans. DREME-GSMR was trained on scan A of P1, and tested on scan B for real-time prediction, using scan B’s dynamic reconstruction as pseudo ‘ground truth’. The top two rows show the SI and AP motion trajectories, with red markers indicating the four time points displayed below. The remaining rows show axial, coronal, and sagittal views of the pseudo ‘ground truth’, DREME-GSMR prediction, and difference images at the selected time points.

Figure 5(a) shows representative SI liver center-of-mass motion trajectories from three volunteers and three patients under cross-scan testing. Overall, the predicted trajectories closely matched the pseudo ‘ground-truth’ motion. Figure 5(b) further presents a representative clinical example. The axial, coronal, and sagittal images at four selected time points showed good visual agreement between the predicted and pseudo ‘ground-truth’ dynamic MRI images.

**Figure 6.**
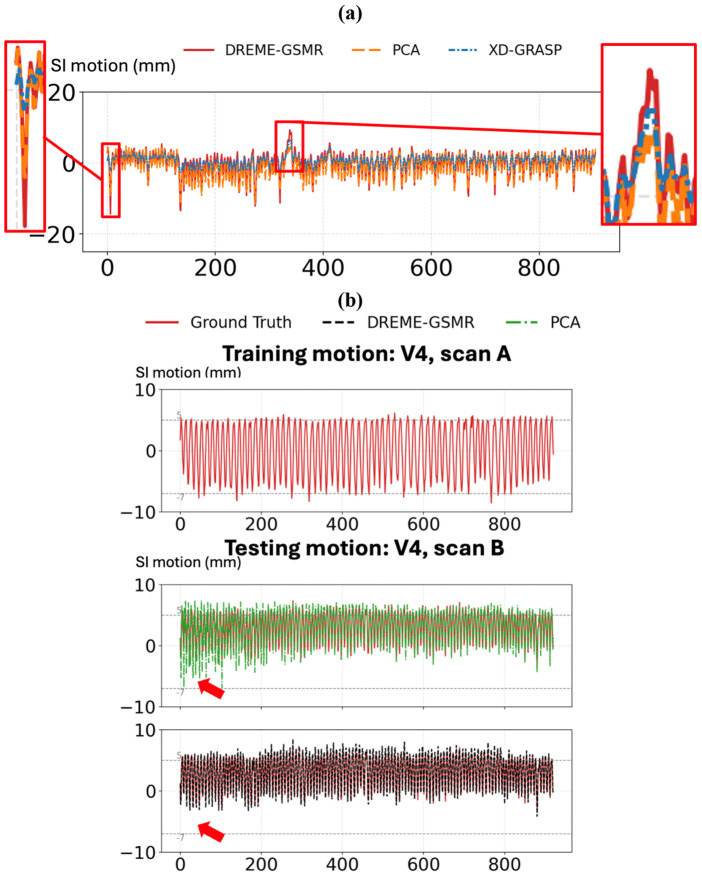
(a) Comparison of liver center-of-mass SI trajectories derived from clinical dynamic reconstruction for case P3. DREME-GSMR, PCA, and XD-GRASP reconstructions from the same scan are overlaid to illustrate the differences in solved motion patterns. (b) Comparison of cross-scan real-time SI motion estimation for V4. Both DREME-GSMR and PCA were trained on scan A and tested on scan B, and the dynamic reconstruction of scan B was used as pseudo ‘ground truth’. The top row shows the training motion from scan A, and the middle and bottom rows show the PCA and DREME-GSMR predictions on scan B, respectively. Horizontal dashed lines indicate reference SI positions

A comparison with XD-GRASP and PCA is summarized in Table IV. Because no ‘ground truth’ was available in the clinical study, XD-GRASP and PCA dynamic reconstructions were compared against the DREME-GSMR dynamic reconstruction from the same scan, which served as the reference. In this subset, PCA generally showed closer agreement with DREME-GSMR than XD-GRASP during dynamic reconstruction, whereas XD-GRASP more frequently underestimated irregular motions, matching observations made in the physical phantom study. This trend is illustrated in Fig. 6(a) for case P3, where PCA remained generally well aligned with DREME-GSMR, while XD-GRASP showed evident motion undershooting. This trend is expected because XD-GRASP relies on phase sorting into a limited number of discrete respiratory states, typically 10 phases. Under irregular breathing, such discretization cannot adequately represent the continuous variation in motion, leading to underestimation of the full motion range.

For real-time imaging, DREME-GSMR and PCA underwent the same cross-scan testing scheme, in which the model was trained on one scan and tested on the paired scan, using the dynamic reconstruction of the test scan as pseudo ‘ground truth’. As shown in Table IV, DREME-GSMR consistently achieved lower real-time localization errors than PCA across all evaluated cases. Figure 6(b) shows one representative example from case V4, where scan A and scan B had large differences in SI motion ranges (10.87±1.05 mm and 4.06±0.78 mm, respectively). In this case, PCA failed to generalize to the unseen motion amplitude (red arrow pointed region), whereas DREME-GSMR remained closely aligned with the pseudo ‘ground-truth’ trajectory, demonstrating better robustness to cross-scan motion variations.

### 5.3 The ablation study results

#### 5.3.1 Encoder design results

To evaluate the benefit of the proposed dual-path motion encoder, we compared DREME-GSMR with two single-encoder variants: DREME-GSMR-MLP and DREME-GSMR-CNN. As summarized in Table V, DREME-GSMR achieved the lowest errors across all phantom and clinical experiments for both dynamic reconstruction and cross-scenario/scan real-time imaging. Figure 8(a) illustrates these findings in the physical phantom study. Although all three models followed the training motion reasonably well, the two single-encoder variants showed reduced robustness on unseen motions, with less accurate recovery of peak amplitudes in the enlarged views. Figure 8(b) shows a representative clinical example of P1 scan B, in which both single-encoder variants exhibited motion undershooting and mismatch relative to the ‘ground truth’, whereas DREME-GSMR remained more closely aligned. Together, these findings support the advantage of combining the MLP-based global motion representation and the CNN-based image-driven motion representation within the progressively trained dual-path framework.

#### 5.3.2 Undersampling study results

To evaluate the robustness of DREME-GSMR to undersampled data, we retrained the model using 75%, 50%, 25%, and 20% of the original radial stacks and compared the results with the full-data model (100%). Three evaluation settings were considered: (1). In the same-scenario/scan dynamic reconstruction, the model was trained and tested on the same scenario/scan data using the specified sampling ratio. (2). In the same-scenario/scan real-time imaging, the model was trained on the sampled portion of a scenario/scan and then used to estimate motion in the remaining frames of that same scenario/scan. Therefore, the 100% condition is not applicable in this setting because no frames remain. (3). In cross-scenario/scan real-time imaging, the model was trained on one scenario/scan using the specified sampling ratio and tested on the fully sampled motion scenario or paired scan.

The quantitative results are summarized in Tables VIII and IX. For the physical phantom study, all errors were computed against the programmed ‘ground-truth’ motion trajectories. As shown in Table VIII, DREME-GSMR maintained good performance from 75% to 25% sampling, with only a gradual degradation in accuracy, whereas a more evident performance drop was observed at 20%. A similar trend was observed for both intra-scenario/scan and cross-scenario/scan real-time imaging, where the 20% condition consistently showed the largest errors. These findings are also shown in Fig. 9(a), where the motion trajectories reconstructed using 25%-75% of the data remain closely aligned with the ‘ground truth’, while the 20% case shows visibly larger mismatch during both dynamic reconstruction and intra-scenario/scan real-time estimation.

For the clinical study, as shown in Table IX, the clinical results followed the same general trend as the phantom study. Model performance remained relatively stable from 75% to 25% sampling but degraded substantially at 20%, particularly for cross-scan testing. This behavior is also demonstrated in Fig. 9(b), where the SI motion trajectories estimated using 25%-100% of the training data show similar patterns, while the 20% trajectory deviates noticeably.

The image-quality results further support these observations. In the phantom study (Fig. 10(a)), the reference-frame images reconstructed with 50% and 75% of the data were visually similar to the full-data reconstruction, whereas the 25% case showed mild degradation, and the 20% case exhibited clear undersampling artifacts. A similar pattern was observed in the clinical subset (Fig. 10(b) and Table VII).

Overall, these results suggest that DREME-GSMR can maintain comparable dynamic reconstruction and real-time motion estimation performance using 25%-50% of the original data, with a clear breakdown emerging at a 20% undersampling ratio. This suggests that the reconstruction under different undersampling ratios are self-consistent. For a typical clinical scan time of ~6 min, this corresponds to a potential reduction in acquisition time to about 1.5-3 min, which may improve patient comfort and facilitate a more efficient treatment workflow.

## 4. Discussion

In this study, we introduced DREME-GSMR, an innovative framework for dynamic volumetric MRI reconstruction and real-time imaging based on Gaussian representations. DREME-GSMR simultaneously reconstructs time-resolved dynamic CBCTs and learns a motion model capable of resolving inter-frame motion directly from a pre-treatment k-space data in a ‘one-shot’ fashion, without requiring predefined anatomical or motion models.

The proposed framework unites three components: a dense 3D-Gaussian reference-frame representation, a hierarchical motion model employing coarse-to-fine MBC Gaussians, and a dual-path motion encoder designed to infer motion scores, to solve the spatiotemporal inverse problem with largely improved computational efficiency. As demonstrated in Tables I-III, DREME-GSMR maintains sub-voxel motion-tracking accuracy while cutting reconstruction time in 70% relative to DREME-MR (from ~200 mins on NVIDIA Tesla V100 to ~140 mins). The switch from implicit neural representations to a sparse Gaussian parameterization also lowers training memory demands: at 2 × 2 × 3 mm resolution, DREME-MR required ~32 GB of GPU memory for a batch size of 32, whereas DREME-GSMR needed only ~22 GB (a ~30 % reduction), which is consistent with the savings we observed previously with the Gaussian-based PMF-STGR (Xie et al., 2025) dynamic-CBCT framework. This memory reduction enables the dynamic reconstruction and real-time imaging framework to run not only on high-end GPUs (e.g., NVIDIA A100) but also on widely available, cost-effective consumer-grade GPUs such as the RTX 4090 commonly found in gaming PCs. By significantly lowering hardware and computational requirements, the framework becomes more accessible to clinics with varying levels of resources, paving the way for broader clinical adoption and ultimately benefiting a larger and more diverse patient population. Beyond speed and memory gains, MBC Gaussians better accommodate organ sliding along body walls, a motion type that challenges B-spline-based MBCs (Shao et al., 2025a). Because the Gaussian kernels are optimized during training, the model adapts naturally to patient-specific motion without the rigidity of pre-defined spline control points. Collectively, these attributes enable DREME-GSMR to deliver robust performance across diverse anatomies and complex motion profiles, making it promising for motion-adaptive radiotherapy.

**Figure 7.**
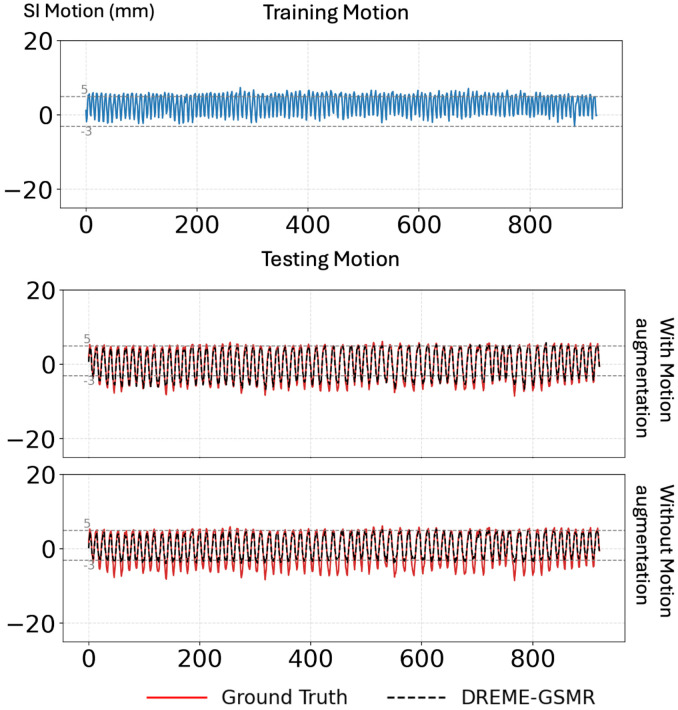
An example illustrating the effect of motion-augmentation training on cross-scan real-time motion estimation. The top row shows the training motion from volunteer V4, scan B, which has a peak-to-valley motion range of 4.06 ± 0.78 mm. The middle and bottom rows show testing on scan A of the same case, which has a motion range of 10.87 ± 1.05 mm, with and without motion augmentation, respectively. The model trained with motion augmentation shows closer agreement with the ‘ground-truth’ trajectory and better recovery of the larger unseen motion amplitude.

**Figure 8.**
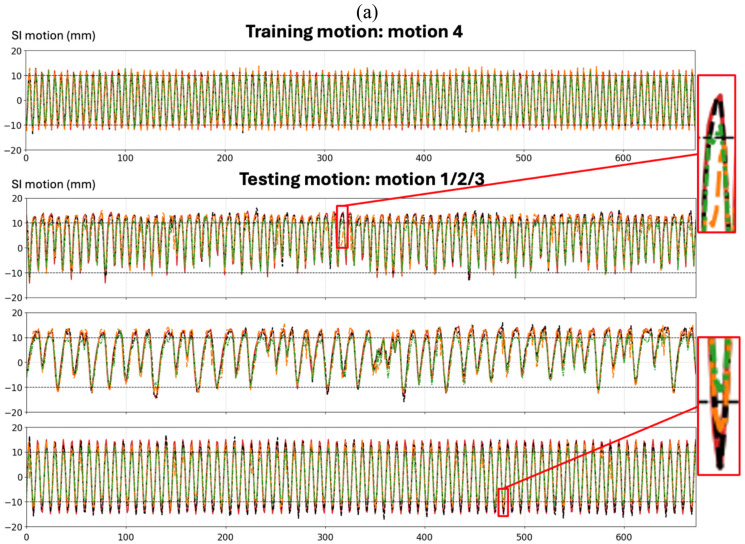

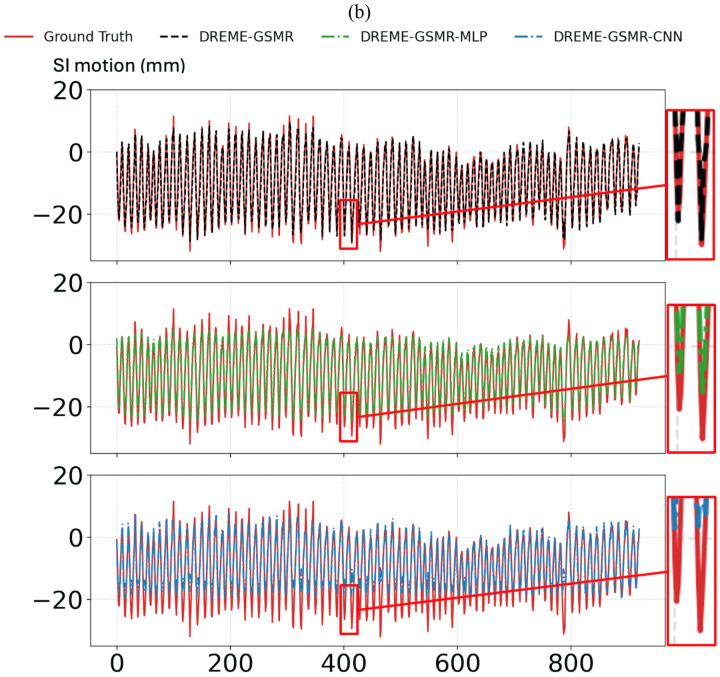
Comparison between DREME-GSMR with two single encoder variants. (a) Comparison of dynamic reconstruction and cross-scenario real-time SI motion trajectories in the ablation phantom study. DREME-GSMR, DREME-GSMR-MLP, and DREME-GSMR-CNN were trained on Motion 4. The top row shows the training motion and dynamic reconstruction results, and the lower three rows show testing on unseen Motions 1-3. Horizontal dashed lines indicate SI positions of -10 and +10 mm. (b) One clinical example of cross-scan real-time motion estimation in the ablation study for case V1 scan B. The ground-truth trajectory is overlaid with the predictions from DREME-GSMR and the two single-encoder variants

**Figure 9.**
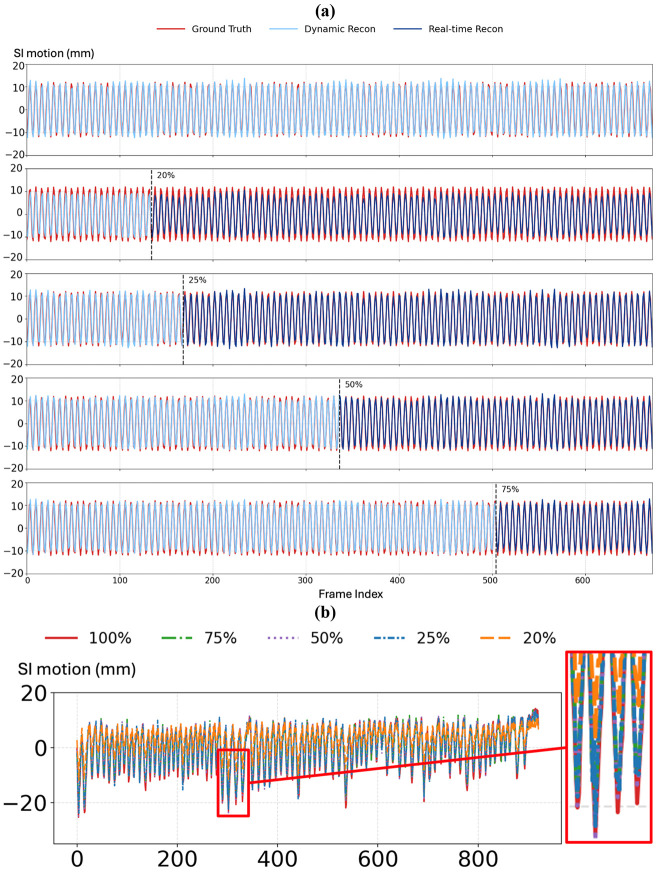
(a) Dynamic reconstruction and intra-scenario real-time SI motion trajectories for physical phantom motion 4 under different undersampling ratios. The top row shows dynamic reconstruction using the full dataset (100%). The lower four rows show models trained using the first 20%, 25%, 50%, and 75% of the scan and then applied to the remaining frames for intra-scenario real-time estimation. Vertical dashed lines indicate the transition point between the training portion and the testing portion of the scan. (b) Comparison of liver SI motion trajectories in the clinical undersampling study for case P1. DREME-GSMR models trained on scan B using 100%, 75%, 50%, 25%, and 20% of the data were tested on the full scan A for cross-scan real-time imaging.

**Figure 10.**
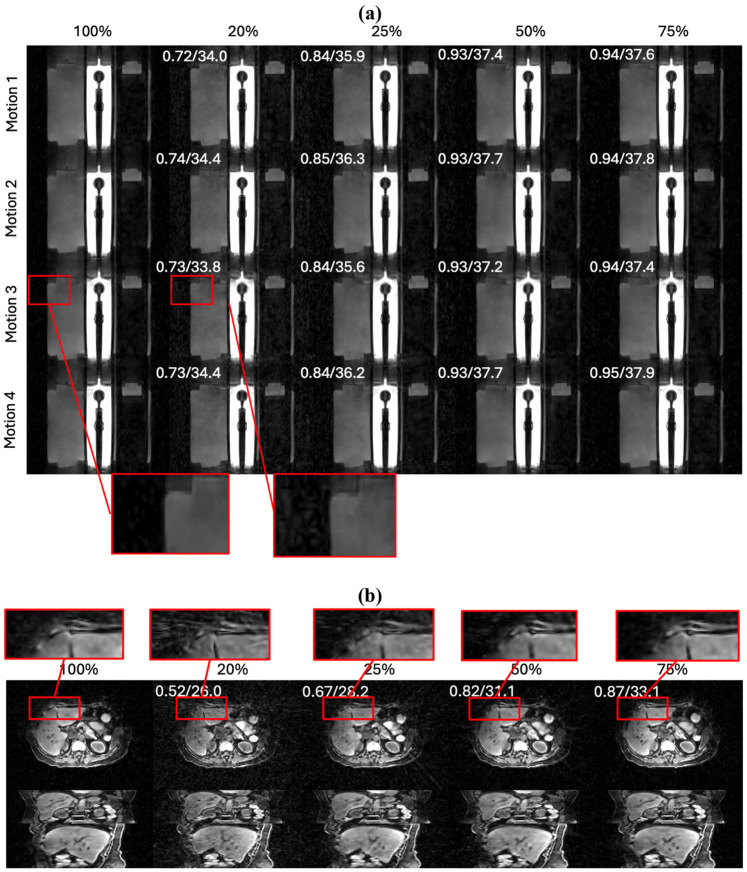
(a) Reference-frame images from the phantom undersampling study using models trained with 100%, 20%, 25%, 50%, and 75% of the radial stack angles. The numbers shown in the upper corner of each undersampled panel denote SSIM/PSNR relative to the corresponding 100% reconstruction. Enlarged views highlight representative undersampling artifacts. (b) Reference-frame images from the clinical undersampling study (P1, scan A) at 100%, 20%, 25%, 50%, and 75% sampling ratios. The numbers displayed on the undersampled panels denote SSIM/PSNR relative to the 100% reconstruction. Enlarged views highlight representative image degradation caused by undersampling.

In DREME-GSMR, we designed a dual-path motion encoder to combine the complementary strengths of MLP-based and CNN-based motion encoders. The MLP branch uses minimal k-space center information to predict temporal motion scores and enables on-the-fly temporal binning during the early training stages, which stabilizes optimization and facilitates coarse-to-fine learning. In contrast, the CNN branch processes 2D FFT projection image inputs and supports motion-augmentation training using simulated data with unseen motion patterns. Our ablation study showed that, under a similar augmentation scheme, the MLP-only variant was less effective than the dual-path models for real-time motion estimation. A likely reason is that the NUFFT-simulated k-space signals used for augmentation are based on very limited k-space data and do not fully reproduce the coil-sensitivity characteristics and noise properties of the acquired data, leading to a gap between the measured and simulated k-space data for the MLP branch. By comparison, the CNN branch can extract motion information from the visual structure of the simulated projections and is therefore more robust to this simulation-to-measurement mismatch. Figure 7 provides a representative example of the effectiveness of motion augmentation training. When the model was trained on a scan with a smaller motion range and tested on a scan with a substantially larger unseen motion range, motion-augmentation training enabled DREME-GSMR to better recover the target trajectory, whereas the model trained without augmentation showed more pronounced motion undershooting. These findings support the role of the CNN branch and motion-augmentation strategy in improving generalizability to unseen motion patterns.

In the future, the training time of DREME-GSMR can be further reduced by employing more advanced hardware (faster GPU cards, for instance, RTX 5090). Additionally, accelerated 3DGS frameworks like FlashGS (Feng et al., 2024) can be integrated into our Gaussian framework to speed up reconstruction fundamentally. We can exploit patient-specific priors to speed up the training. Our recently introduced DREME-adapt framework (Zuo et al., 2025) performs a one-time ‘virtual-fraction’ reconstruction from the pre-treatment 4D-CT, then warm-starts subsequent fractions with the reference CBCT and motion model solved by the preceding fraction in a daisy chain fashion, which has demonstrated an 85% reduction in training time in our initial tests while maintaining reconstruction and motion tracking accuracy. Practically, with advanced hardware, using DREME-adapt for DREME-GSMR could cut the reconstruction time from ~140 minutes down to 5~10 minutes, which would potentially meet clinical timing requirements.

## 5. Conclusion

In this study, we present DREME-GSMR, a novel framework for time-resolved dynamic MRI reconstruction and real-time motion management based on 3D Gaussian representations. Leveraging the strong representation power of Gaussians, DREME-GSMR enables ‘one-shot’ dynamic MRI reconstruction directly from raw k-space data, eliminating the need for prior anatomical or motion models. The learned dynamic models further enable subsequent real-time imaging using minimal k-space data. Compared to the original DREME-MR, our approach delivers higher-quality reconstructions with reduced noise, comparable or improved motion tracking accuracy, lower GPU memory demands, and approximately 30% faster training, enhancing its clinical practicality. Furthermore, we performed comprehensive validation on digital phantom, physical phantom, and clinical datasets, demonstrating the robustness, generalizability, and strong overall performance of the proposed framework across controlled and realistic imaging scenarios. Overall, DREME-GSMR advances the clinical feasibility of time-resolved volumetric MRI for motion-adaptive radiotherapy and other motion-sensitive treatments.

## Figures and Tables

**Table I.** Accuracy of solved dynamic MRIs and the corresponding motion for the XCAT study. The tasks of dynamic reconstruction and real-time imaging are separately evaluated. The results are presented as the mean ± standard deviation. Better values are in bold. The arrows are pointing in the direction of higher accuracy.

Method	Same-scenario dynamic reconstruction		
	DSC↑	SSIM↑	COME (mm)↓
DREME-MR	**0.92±0.02**	0.84±0.02	0.66±0.21
DREME-GSMR	**0.92±0.02**	**0.92±0.01**	**0.50±0.15**
Method	Cross-scenario real-time imaging		
	DSC↑	SSIM↑	COME (mm)↓
DREME-MR	0.91±0.02	0.82±0.02	0.74±0.27
DREME-GSMR	**0.92±0.03**	**0.91±0.02**	**0.65±0.19**

**Table II.** COME of the spherical tracking target for same-scenario dynamic reconstruction and cross-scenario real-time imaging in the physical phantom study. Results are reported as mean ± standard deviation (mm). Better values are in bold.

Motion Scenarios	Same-scenario dynamic reconstruction		
	DREME-GSMR	XD-GRASP	PCA
Motion 1→1	**1.03±1.04**	4.71±3.61	2.90±2.07
Motion 2→2	**0.87±0.71**	3.29±2.18	2.07±1.54
Motion 3→3	**1.52±0.86**	2.08±1.35	3.15±1.83
Motion 4→4	**1.36±0.96**	2.08±1.63	1.88±1.06
Motion Scenarios	Cross-scenario real-time imaging		
	DREME-GSMR	XD-GRASP	PCA
Motion 1→2,3,4	**1.41±1.16**	N/A	3.45±2.22
Motion 2→1,3,4	**1.73±1.38**	N/A	3.84±2.36
Motion 3→1,2,4	**1.27±1.02**	N/A	3.43±2.30
Motion 4→1,2,3	**1.41±1.16**	N/A	2.92±1.89

**Table III.** Average peak-to-valley respiratory motion range and cross-scan real-time liver localization error in the clinical dataset. Results are reported separately for healthy volunteers and patients and are presented as mean ± standard deviation (mm).

Dataset	Motion Range (mm)			Localization Error (mm)			
	SI	AP	LR	3D	SI	AP	LR
Volunteer	10.26±5.61	3.33±2.03	0.91±0.58	1.31±0.82	0.83±0.74	0.57±0.57	0.47±0.40
Patient	4.47±2.63	1.98±1.43	0.56±0.36	0.96±0.64	0.60±0.53	0.49±0.50	0.33±0.31

**Table IV.** Comparison of DREME-GSMR, XD-GRASP, and PCA for clinical dynamic reconstruction and cross-scan real-time imaging. Results are reported as mean ± standard deviation (mm).

Case ID	Scan ID	Same-scan dynamic reconstruction		
		DREME-GSMR	XD-GRASP	PCA
V1	A→A	N/A	2.23±1.34	1.98±1.11
	B→B	N/A	3.11±1.13	2.55±1.40
V4	A→A	N/A	2.68±1.68	1.76±0.92
	B→B	N/A	1.28±0.67	1.20±0.51
P1	A→A	N/A	1.31±0.59	1.35±0.51
	B→B	N/A	1.21±0.59	1.13±0.54
P3	A→A	N/A	1.73±1.47	1.62±0.99
	B→B	N/A	2.02±2.06	1.03±0.53
Case ID	Scan ID	Cross-scan real-time imaging		
		DREME-GSMR	XD-GRASP	PCA
V1	A→B	2.11±1.22	N/A	3.17±1.61
	B→A	2.20±1.07	N/A	3.52±1.86
V4	A→B	1.26±0.54	N/A	1.95±0.82
	B→A	1.33±0.54	N/A	2.51±1.39
P1	A→B	0.69±0.33	N/A	1.27±0.73
	B→A	0.80±0.40	N/A	1.36±0.58
P3	A→B	0.23±0.20	N/A	1.02±0.65
	B→A	0.23±0.27	N/A	1.07±0.99

**Table V.** Comparison of DREME-GSMR and the two single-encoder variants in the phantom ablation study. Results are reported as COME in mean ± standard deviation (mm) for dynamic reconstruction and cross-scenario real-time imaging. Lower values indicate better performance.

Motion Scenarios	Same-scenario dynamic reconstruction		
	DREME-GSMR	DREME-GSMR-MLP	DREME-GSMR-CNN
Motion 1→1	**1.03±1.04**	2.11±1.71	1.33±1.15
Motion 2→2	**0.87±0.71**	1.35±1.26	0.93±0.97
Motion 3→3	**1.52±0.86**	2.89±1.58	1.63±1.22
Motion 4→4	**1.36±0.96**	1.94±1.21	1.49±0.92
Motion Scenarios	Cross-scenario real-time imaging		
	DREME-GSMR	DREME-GSMR-MLP	DREME-GSMR-CNN
Motion 1→2,3,4	**1.41±1.16**	2.35±1.56	1.90±1.95
Motion 2→1,3,4	**1.73±1.38**	2.49±1.72	1.84±1.77
Motion 3→1,2,4	**1.27±1.02**	2.25±1.59	1.71±1.39
Motion 4→1,2,3	**1.41±1.16**	2.49±1.48	1.82±1.82

**Table VI.** Comparison of DREME-GSMR and the two single-encoder variants for cross-scan real-time imaging in the clinical ablation subset. Results are reported as mean ± standard deviation (mm). Lower values indicate better performance.

Case ID	Scan ID	Cross-scan real-time imaging		
		DREME-GSMR	DREME-GSMR-MLP	DREME-GSMR-CNN
V1	A→B	**2.11±1.22**	2.50±0.99	2.89±1.07
	B→A	**2.20±1.07**	4.15±1.15	4.97±3.93
V4	A→B	**1.26±0.54**	2.43±0.65	2.43±0.63
	B→A	**1.33±0.54**	1.60±0.79	1.57±0.68
P1	A→B	**0.69±0.33**	0.77±0.31	1.10±0.59
	B→A	**0.80±0.40**	0.84±0.41	0.83±0.44
P3	A→B	**0.23±0.20**	1.02±0.65	1.16±0.92
	B→A	**0.23±0.27**	1.16±1.12	1.35±1.40

**Table VII.** SSIM and PSNR of clinical reference-frame images reconstructed under different undersampling ratios, using the full-acquisition reconstruction as reference. Results are reported for the clinical subset cases V1, V4, P1, and P3 as mean ± standard deviation.

Undersample ratio	SSIM	PSNR
20%	0.61±0.08	29.06±2.52
25%	0.75±0.06	31.05±2.13
50%	0.86±0.03	33.30±1.71
75%	0.89±0.02	34.92±1.61

**Table VIII.** Physical phantom undersampling study results. Errors are reported as mean ± standard deviation (mm) for dynamic reconstruction, intra-scenario real-time imaging, and cross-scenario real-time imaging under different sampling ratios. Lower values indicate better performance.

Motion Scenarios	Same-scenario dynamic reconstruction				
	100%	75%	50%	25%	20%
Motion 1→1	1.03±1.04	1.20±1.30	1.48±1.67	1.57±1.70	1.70±1.93
Motion 2→2	0.87±0.71	0.90±0.84	1.16±1.17	1.14±1.21	1.61±1.39
Motion 3→3	1.52±0.86	1.61±0.72	1.61±0.76	1.64±0.92	2.09±1.14
Motion 4→4	1.36±0.96	1.38±0.99	1.40±0.78	1.44±0.76	1.96±1.16
Motion Scenarios	Intra-scenario real-time imaging				
	100%	75%	50%	25%	20%
Motion 1→1	N/A	1.42±1.36	1.50±1.52	1.70±1.64	1.83±1.87
Motion 2→2	N/A	0.98±1.02	1.22±1.32	1.29±1.24	1.81±1.41
Motion 3→3	N/A	1.70±0.97	1.67±1.00	1.88±1.16	2.70±1.28
Motion 4→4	N/A	1.46±0.91	1.47±0.97	1.62±0.93	2.21±1.12
Motion Scenarios	Cross-scenario real-time imaging				
	100%	75%	50%	25%	20%
Motion 1→2,3,4	1.41±1.16	1.97±1.88	2.30±2.22	2.33±2.31	2.87±2.80
Motion 2→1,3,4	1.73±1.38	1.80±1.71	2.42±2.29	2.43±2.08	2.84±2.03
Motion 3→1,2,4	1.27±1.02	1.38±0.72	1.41±0.83	1.43±0.98	1.76±1.08
Motion 4→1,2,3	1.41±1.16	1.47±0.97	1.53±1.04	1.57±1.03	2.61±1.63

**Table IX.** Clinical undersampling study results for a subset of cases. For same-scan dynamic reconstruction and intra-scan real-time imaging, the undersampled models were compared against the corresponding full-data reconstruction from the same scan. For cross-scan real-time imaging, the dynamic reconstruction of the paired scan was used as pseudo ground truth. Errors are reported as mean ± standard deviation (mm), with lower values indicating better performance.

Case ID	Scan ID	Same-scan dynamic reconstruction				
		100%	75%	50%	25%	20%
V1	A→A	N/A	1.62±0.62	1.72±0.99	1.94±1.03	2.25±1.23
	B→B	N/A	1.05±0.70	2.06±0.72	1.98±0.97	4.55±2.66
V4	A→A	N/A	0.95±0.41	1.09±0.55	1.27±0.39	1.98±0.65
	B→B	N/A	1.23±0.64	1.01±0.50	1.46±0.55	2.32±0.71
P1	A→A	N/A	0.84±0.32	0.91±0.29	1.44±0.39	1.48±0.36
	B→B	N/A	0.69±0.27	0.83±0.40	1.26±0.45	2.83±0.44
P3	A→A	N/A	1.07±0.51	1.44±0.81	1.60±0.80	1.63±0.99
	B→B	N/A	0.91±0.65	0.92±0.66	0.87±0.63	1.64±0.71
Case ID	Scan ID	Intra-scan real-time imaging				
		100%	75%	50%	25%	20%
V1	A→A	N/A	1.93±0.70	1.95±1.15	2.16±0.98	2.54±1.43
	B→B	N/A	1.12±0.70	2.09±0.86	2.14±1.07	5.15±2.80
V4	A→A	N/A	1.07±0.47	1.61±0.66	1.43±0.56	2.16±0.83
	B→B	N/A	1.31±0.71	1.23±0.59	1.88±0.60	2.82±0.86
P1	A→A	N/A	0.88±0.32	1.01±0.37	1.51±0.33	1.55±0.41
	B→B	N/A	0.70±0.29	1.07±0.51	1.55±0.51	3.03±0.44
P3	A→A	N/A	1.14±0.61	1.55±1.04	1.71±0.86	2.05±1.14
	B→B	N/A	0.93±0.52	0.96±0.73	1.03±0.88	1.78±0.75
Case ID	Scan ID	Cross-scan real-time imaging				
		100%	75%	50%	25%	20%
V1	A→B	2.11±1.22	2.22±1.00	2.31±1.12	2.52±1.19	5.63±3.14
	B→A	2.20±1.07	2.52±1.36	3.75±2.71	3.76±1.72	5.10±2.73
V4	A→B	1.26±0.54	1.55±0.75	1.78±0.57	1.81±0.66	2.05±1.66
	B→A	1.33±0.54	1.35±0.61	1.40±0.57	1.47±0.53	1.66±0.65
P1	A→B	0.69±0.33	0.89±0.32	1.23±0.53	1.57±0.52	2.38±0.42
	B→A	0.80±0.40	0.81±0.36	1.10±0.45	1.55±0.44	1.84±0.47
P3	A→B	0.23±0.20	0.98±0.50	1.00±0.59	1.11±0.68	1.66±0.64
	B→A	0.23±0.27	1.47±0.97	1.69±1.08	1.88±1.50	1.69±1.08

## Data Availability

The UTSW patient dataset was retrospectively collected in 2025 and 2026 from an approved study at UT Southwestern Medical Center, under an umbrella IRB protocol 082013-008 (Improving radiation treatment quality and safety by retrospective data analysis). The volunteer dataset was acquired at UTSW by a separate IRB protocol (STU2021-0206). This is an analysis study and not a clinical trial. Individual volunteer/patient consent was signed for the anonymized use of the imaging data for analysis. Our in-house dataset is not publicly available because the data includes sensitive and confidential patient information. Physical phantom data is available at link.

## Appendix

**Table A.1.** Per-case peak-to-valley respiratory motion range and cross-scan real-time liver localization error in the clinical dataset. Results are reported as mean ± standard deviation (mm)

Case ID	Scan ID	Motion range (mm)				Localization Error (mm)			
		SI	AP	LR	3D	SI	AP	LR
V1	A	16.31 ± 4.25	5.77 ± 1.98	0.41 ± 0.34	2.11 ± 1.22	1.54 ± 1.02	1.11 ± 0.93	0.31 ± 0.33
	B	23.32 ± 4.34	7.96 ± 1.39	0.38 ± 0.24	2.20 ± 1.07	1.50 ± 1.15	1.14 ± 0.70	0.29 ± 0.37
V2	A	9.40 ± 3.14	1.91 ± 0.61	1.38 ± 0.41	1.08 ± 0.62	0.68 ± 0.49	0.59 ± 0.56	0.34 ± 0.28
	B	10.82 ± 3.73	2.61 ± 1.10	1.26 ± 0.46	1.84 ± 0.75	1.36 ± 0.68	0.90 ± 0.70	0.48 ± 0.35
V3	A	8.24 ± 1.68	2.62 ± 0.58	1.13 ± 0.36	1.07 ± 0.47	0.58 ± 0.46	0.40 ± 0.27	0.62 ± 0.44
	B	8.57 ± 2.35	2.91 ± 0.78	1.37 ± 0.38	1.18 ± 0.54	0.65 ± 0.53	0.44 ± 0.31	0.67 ± 0.48
V4	A	10.87 ± 1.05	4.47 ± 0.54	2.03 ± 0.28	1.26 ± 0.54	0.69 ± 0.51	0.33 ± 0.24	0.85 ± 0.50
	B	4.06 ± 0.78	1.94 ± 0.37	0.70 ± 0.30	1.33 ± 0.54	0.74 ± 0.50	0.32 ± 0.24	0.65 ± 0.31
V5	A	5.52 ± 1.09	1.66 ± 0.43	0.63 ± 0.23	0.84 ± 0.46	0.52 ± 0.45	0.36 ± 0.32	0.37 ± 0.25
	B	6.08 ± 1.30	1.81 ± 0.54	0.54 ± 0.19	0.96 ± 0.41	0.62 ± 0.42	0.42 ± 0.33	0.42 ± 0.28
V6	A	9.31 ± 1.55	3.32 ± 0.62	0.57 ± 0.16	0.86 ± 0.45	0.54 ± 0.44	0.42 ± 0.33	0.31 ± 0.25
	B	10.42 ± 1.88	2.85 ± 0.51	0.53 ± 0.17	0.89 ± 0.46	0.55 ± 0.48	0.41 ± 0.34	0.34 ± 0.28
P1	A	6.36 ± 1.67	2.56 ± 0.71	0.39 ± 0.13	0.69 ± 0.33	0.45 ± 0.33	0.27 ± 0.20	0.31 ± 0.24
	B	6.21 ± 1.54	2.56 ± 0.66	0.38 ± 0.19	0.80 ± 0.40	0.59 ± 0.43	0.25 ± 0.19	0.34 ± 0.24
P2	A	1.61 ± 0.45	1.86 ± 0.52	0.24 ± 0.10	0.86 ± 0.41	0.47 ± 0.33	0.58 ± 0.40	0.22 ± 0.19
	B	1.95 ± 0.53	1.71 ± 0.54	0.27 ± 0.11	0.83 ± 0.39	0.41 ± 0.33	0.57 ± 0.39	0.23 ± 0.20
P3	A	4.72 ± 2.46	1.90 ± 0.93	0.61 ± 0.16	0.23 ± 0.20	0.15 ± 0.16	0.09 ± 0.09	0.10 ± 0.13
	B	4.11 ± 4.75	2.55 ± 2.39	0.69 ± 0.14	0.23 ± 0.27	0.15 ± 0.22	0.10 ± 0.15	0.09 ± 0.12
P4	A	3.04 ± 0.74	3.07 ± 0.82	1.04 ± 0.28	0.82 ± 0.70	0.49 ± 0.48	0.53 ± 0.54	0.26 ± 0.22
	B	2.40 ± 0.84	2.20 ± 0.62	0.58 ± 0.27	0.79 ± 0.67	0.47 ± 0.46	0.51 ± 0.52	0.27 ± 0.21
P5	A	2.45 ± 1.02	0.79 ± 0.17	0.21 ± 0.25	0.79 ± 0.44	0.65 ± 0.48	0.24 ± 0.18	0.19 ± 0.16
	B	2.59 ± 1.20	0.61 ± 0.25	0.35 ± 0.25	0.71 ± 0.38	0.56 ± 0.41	0.24 ± 0.17	0.21 ± 0.17
P6	A	7.33 ± 1.20	1.78 ± 1.00	0.63 ± 0.47	0.90 ± 0.46	0.54 ± 0.41	0.49 ± 0.36	0.35 ± 0.30
	B	6.07 ± 0.98	1.79 ± 1.19	0.53 ± 0.29	1.03 ± 0.55	0.63 ± 0.53	0.52 ± 0.37	0.39 ± 0.34
P7	A	3.30 ± 0.87	1.24 ± 0.11	0.31 ± 0.23	0.95 ± 0.54	0.62 ± 0.57	0.44 ± 0.32	0.26 ± 0.27
	B	5.60 ± 1.02	1.74 ± 0.25	0.56 ± 0.28	1.05 ± 0.49	0.61 ± 0.51	0.55 ± 0.38	0.41 ± 0.30
P8	A	2.89 ± 1.46	1.07 ± 0.56	0.35 ± 0.53	1.10 ± 0.61	0.64 ± 0.56	0.63 ± 0.50	0.27 ± 0.29
	B	2.76 ± 0.62	0.94 ± 0.73	0.25 ± 0.26	1.00 ± 0.51	0.57 ± 0.44	0.57 ± 0.45	0.23 ± 0.28
P9	A	1.48 ± 0.66	0.92 ± 0.37	0.55 ± 0.28	0.74 ± 0.48	0.57 ± 0.44	0.29 ± 0.22	0.30 ± 0.21
	B	1.55 ± 0.42	0.86 ± 0.23	0.41 ± 0.17	0.54 ± 0.26	0.37 ± 0.25	0.25 ± 0.19	0.20 ± 0.16
P10	A	2.54 ± 2.47	1.44 ± 0.65	0.29 ± 0.17	1.28 ± 0.80	0.71 ± 0.37	0.86 ± 0.83	0.22 ± 0.24
	B	4.30 ± 3.37	1.45 ± 0.36	0.69 ± 0.23	1.36 ± 0.90	0.80 ± 0.42	0.88 ± 0.93	0.37 ± 0.26
P11	A	5.50 ± 1.43	1.48 ± 1.82	0.52 ± 0.23	1.51 ± 0.95	1.00 ± 0.81	0.88 ± 0.75	0.35 ± 0.27
	B	5.02 ± 1.11	1.68 ± 1.60	0.44 ± 0.17	1.66 ± 0.95	1.16 ± 0.89	0.94 ± 0.67	0.36 ± 0.25
P12	A	6.51 ± 1.39	2.37 ± 0.63	0.20 ± 0.05	0.90 ± 0.73	0.72 ± 0.69	0.40 ± 0.38	0.16 ± 0.13
	B	6.79 ± 1.02	2.42 ± 0.54	0.29 ± 0.10	1.01 ± 0.74	0.86 ± 0.71	0.38 ± 0.38	0.20 ± 0.13
P13	A	7.65 ± 3.97	3.00 ± 1.18	0.41 ± 0.15	1.21 ± 0.74	0.70 ± 0.5	0.79 ± 0.67	0.25 ± 0.17
	B	3.43 ± 0.72	3.36 ± 0.81	0.38 ± 0.13	1.09 ± 0.52	0.63 ± 0.49	0.69 ± 0.51	0.25 ± 0.18
P14	A	4.72 ± 1.19	1.71 ± 0.82	0.71 ± 0.16	0.88 ± 0.44	0.51 ± 0.41	0.32 ± 0.26	0.51 ± 0.34
	B	4.37 ± 1.04	1.52 ± 0.68	0.78 ± 0.12	0.93 ± 0.45	0.56 ± 0.44	0.35 ± 0.28	0.51 ± 0.33
P15	A	3.21 ± 1.16	1.51 ± 0.65	0.57 ± 0.17	1.10 ± 0.81	0.64 ± 0.57	0.88 ± 0.76	0.43 ± 0.28
	B	4.46 ± 1.17	1.30 ± 1.05	0.43 ± 0.19	1.18 ± 0.62	0.56 ± 0.44	0.78 ± 0.64	0.32 ± 0.30
P16	A	3.54 ± 0.95	1.06 ± 0.56	1.03 ± 0.31	1.19 ± 0.63	0.55 ± 0.44	0.38 ± 0.31	0.85 ± 0.59
	B	2.35 ± 0.54	1.51 ± 0.31	0.98 ± 0.23	1.23 ± 0.62	0.53 ± 0.44	0.47 ± 0.36	0.85 ± 0.60
P17	A	7.26 ± 1.98	2.46 ± 0.85	0.80 ± 0.23	1.09 ± 0.53	0.69 ± 0.54	0.62 ± 0.41	0.33 ± 0.23
	B	7.46 ± 2.39	2.16 ± 0.77	0.85 ± 0.29	1.14 ± 0.62	0.74 ± 0.69	0.61 ± 0.37	0.31 ± 0.24
P18	A	9.07 ± 2.06	5.83 ± 1.45	0.60 ± 0.26	0.91 ± 0.54	0.62 ± 0.52	0.42 ± 0.39	0.28 ± 0.21
	B	8.38 ± 2.37	5.96 ± 1.68	0.73 ± 0.31	0.83 ± 0.45	0.55 ± 0.43	0.38 ± 0.38	0.28 ± 0.21
P19	A	3.87 ± 1.35	2.11 ± 0.74	0.91 ± 0.13	0.86 ± 0.50	0.56 ± 0.45	0.33 ± 0.32	0.40 ± 0.32
	B	3.24 ± 1.24	1.64 ± 0.63	0.88 ± 0.22	0.91 ± 0.60	0.62 ± 0.53	0.37 ± 0.38	0.39 ± 0.32
P20	A	4.41 ± 1.15	1.92 ± 0.64	0.78 ± 0.70	0.95 ± 0.47	0.57 ± 0.45	0.46 ± 0.38	0.40 ± 0.29
	B	4.12 ± 1.06	1.02 ± 0.45	0.62 ± 0.54	1.03 ± 0.53	0.65 ± 0.52	0.47 ± 0.41	0.40 ± 0.31
